# Cost-effectiveness of adding enzalutamide vs standard non-steroidal antiandrogen drugs for patients with metastatic hormone-sensitive prostate cancer undergoing standard first-line therapy: a within-trial, secondary end-point analysis of the ENZAMET phase 3, international randomized trial (ANZUP 1304)

**DOI:** 10.1016/j.eclinm.2026.104207

**Published:** 2026-10-05

**Authors:** Chi Kin Law, Martin R. Stockler, Andrew J. Martin, Ian D. Davis, Christopher J. Sweeney, Rachael L. Morton

**Affiliations:** a Sydney Clinical Trials Centre, The University of Sydney, Camperdown, New South Wales, Australia; b Faculty of Health, Medicine and Behavioural Sciences, UQ Centre for Clinical Research, Royal Brisbane & Women's Hospital Campus, University of Queensland, Herston, Queensland, Australia; c Eastern Health Clinical School, Monash University, Box Hill, Victoria, Australia; d South Australian Immunogenomics Cancer Institute, University of Adelaide, Adelaide, South Australia, Australia

**Keywords:** Cost-effectiveness analysis, Prostatic neoplasms, Androgen antagonists, Neoplasm metastasis, Healthcare costs

## Abstract

**Background:**

The ENZAMET randomized trial demonstrated adding enzalutamide to testosterone suppression, compared with a less potent comparator, significantly improved overall and progression-free survival in men with metastatic hormone-sensitive prostate cancer (mHSPC). This study evaluated cost-effectiveness of adding enzalutamide to standard therapy over 60 months.

**Methods:**

A within-trial cost-effectiveness analysis was conducted from an Australian health system perspective using prospectively collected, observed data from ENZAMET open-label, phase 3 RCT. This study is registered with ClinicalTrials.gov, NCT02446405, ANZCTR, ACTRN12614000110684, and EudraCT, 2014-003190-42. The analysis was conducted utilizing a data cut-off date of January 19, 2022, with a median follow-up of 68 months. Information on disease status, healthcare utilization, and health-related quality of life were extracted from trial records, linked Medicare claims and patient questionnaires. Costs were calculated in 2023 Australian dollars. EQ-5D-5L scores were converted to health utilities using a validated algorithm. Quality-adjusted life years (QALYs) were estimated by multiplying mean health utility with corresponding life-years accumulated. Deterministic and probabilistic sensitivity analyses examined the effect of varying unit price of study drugs, per diem rates of hospital stays, benefits paid from Medicare claims, and health utility values on robustness of results. Data were assessed for missingness and imputation applied where relevant.

**Findings:**

Enzalutamide was associated with an additional AUD$127,262 per patient in healthcare costs, 0.24 life-years and 0.15 QALYs gained in the first 5 years, compared with non-steroidal antiandrogen therapy. The incremental cost-effectiveness ratio, (ICER) was AUD$543,880 per life-year gained and AUD$835,580 per QALY gained. Results were sensitive to the cost of study drug, with a purchase price of AUD$6.73 per tablet yielding an ICER that met the willingness-to-pay threshold of AUD$50,000 per QALY gained. Of note, hospitalizations were slightly higher in the enzalutamide group; as was use of musculoskeletal medicines, the latter indicating falls and fractures.

**Interpretation:**

Although Enzalutamide improves quality-adjusted survival for men with mHSPC, it is unlikely to be cost-effective. Long-term bone health management in survivors is an important clinical consideration.

**Funding:**

Astellas Pharma and Australian National Health and Medical Research Council.

**Research in context**

**Evidence before this study**

We searched MEDLINE, Embase, EBM Reviews–NHS Economic Evaluation and Health Technology Assessment database via Ovid, from database inception to July 22, 2026, for papers published in English, using the terms “metastatic hormone-sensitive prostate cancer or mHSPC”, “enzalutamide” and “economic evaluation or cost-effectiveness”. Our search yielded 37 results. Adding enzalutamide to testosterone suppression as first-line androgen deprivation therapy for metastatic hormone-sensitive prostate cancer (mHSPC) significantly increased overall survival. The drug was listed for reimbursement by the Australian Pharmaceutical Benefits Scheme in 2023 after a submission showing the drug was potentially cost-effective over a 15-year time horizon. However, this relied on modeled evidence with limited use of prospective data from a within-trial cost-effectiveness analysis.

**Added value of this study**

Compared with non-steroidal antiandrogen, enzalutamide was associated with higher costs and greater quality-adjusted life year (QALY) gains over the first five years. However, at current market rates, the within-trial incremental cost-effectiveness ratio exceeded Australia's implicit cost-effectiveness threshold. Enzalutamide could be cost-effective with lower drug pricing. Longer-term enzalutamide use was also associated with an increased risk of falls and bone fractures, leading to higher healthcare costs in this population.

**Implications of all the available evidence**

The higher healthcare costs associated with enzalutamide treatment are driven by drug pricing, a longer duration of therapy, and possible treatment-related complications such as falls and fractures. The latter are potentially preventable and, if well managed, could enhance the economic and clinical benefits associated with longer progression-free and overall survival.

## Introduction

Prostate cancer is the most commonly diagnosed malignant tumor in men, with about 10% of patients expected to develop distant metastases either after being diagnosed with localized disease or as the initial presentation of the disease.^1^, ^2^, ^3^ Enzalutamide is a more effective oral androgen receptor pathway inhibitor (ARPI), than earlier types of non-steroidal antiandrogens (NSAA) such as bicalutamide, flutamide, or nilutamide.^4^, ^5^, ^6^ The ENZAMET randomized trial (ANZUP 1304, NCT02446405) was led by the Australian and New Zealand Urogenital and Prostate Cancer Trials Group (ANZUP) and the National Health and Medical Research Council (NHMRC) Clinical Trials Centre, sponsored by the University of Sydney, showed that addition of enzalutamide to testosterone suppression as first-line androgen deprivation therapy for metastatic hormone-sensitive prostate cancer (mHSPC) significantly increased both overall survival (HR,0.70, 95% CI, 0.58–0.84) and progression-free survival (HR, 0.45; 95% CI, 0.39–0.53) compared with NSAA,^7^^,^^8^ with benefits maintained up to 5 years.

Limited health economic assessments in mHSPC have been published despite the known clinical effectiveness of enzalutamide in this setting.^9^, ^10^, ^11^ The incremental cost-effectiveness ratios (ICERs) of adding enzalutamide to androgen deprivation therapy (ADT) for mHSPC were reported at $430,934 per quality-adjusted life year (QALY) gained in the United States (US) and $225,445 in China, both significantly exceeding standard payer thresholds.^9^ These results proved robust in sensitivity analyses, with the high cost of enzalutamide and health utility assumptions identified as the primary economic drivers.^9^ Furthermore, when compared directly to abiraterone plus ADT in the US, enzalutamide produced a very high ICER of $509,813 per QALY gained.^10^ Consequently, research suggests enzalutamide would only achieve cost-effectiveness in the US market if a minimum price discount of 47% were applied.^11^

Because previous economic evaluations have consistently shown enzalutamide is unlikely to be cost-effective at current pricing, health technology assessment (HTA) agencies rarely grant unrestricted reimbursement for metastatic hormone-sensitive prostate cancer (mHSPC). This challenge is clearly reflected in a recent patient access landscape review conducted by ESMO: only five of the 23 HTA agencies in Europe provided unrestricted reimbursement for mHSPC, while three offered reimbursement with restrictions.^12^ Enzalutamide for the treatment of mHSPC was listed for reimbursement by the Australian Pharmaceutical Benefits Scheme in 2023^13^ following a submission reporting it was potentially cost-effective over a 15-year modeled time horizon, with a submitted ICER of $35,000–$45,000 per QALY gained.^14^ The cost of enzalutamide varies greatly around the world, with the most notable differences being between countries that negotiate price as part of universal health care (e.g. United Kingdom [UK], Australia); compared with those where negotiations take place with private insurers.

This analysis aimed to assess the within-trial cost-effectiveness of enzalutamide therapy and to determine the maximum price per tablet required to meet the willingness-to-pay threshold for patients with mHSPC, based on prospective data from the ENZAMET trial over a 5-year period, from an Australian health system perspective. This analysis included the majority of healthcare costs related to the consequences of the treatment and its medium-term management.

## Methods

### Study participants

Our analysis includes all participants from ENZAMET, an open-label, phase 3 randomized controlled trial assessing the addition of enzalutamide to testosterone suppression compared with a conventional NSAA plus testosterone suppression as first-line therapy for mHSPC.^7^^,^^8^ Docetaxel treatment was also permitted at clinician discretion. This trial was registered with ClinicalTrials.gov, NCT02446405, ANZCTR, ACTRN12614000110684, and EudraCT, 2014-003190-42. Between March 2014 and March 2017, 1125 participants from 83 trial sites in Australia, Canada, Ireland, New Zealand, the UK, and the US (Figure S1 in Supplementary Material), were randomized (1:1) to treatment with either enzalutamide or a conventional NSAA. Randomization was stratified by volume of disease, planned use of early docetaxel, planned use of a bone antiresorptive therapy, Adult Comorbidity Evaluation score, and trial site. Cost-effectiveness (health outcomes relative to costs) for the addition of enzalutamide to testosterone suppression in mHSPC was an *a priori* secondary endpoint (Secondary outcome 5) at trial registration.^8^ Details of the trial design and key findings have been published previously.^7^^,^^8^

### Ethics statement

The ENZAMET was conducted in accordance with the principles of Good Clinical Practice (GCP) guidelines and the Declaration of Helsinki. Participants gave written, informed consent before randomization and ethics approval (HREC/13/RPAH/558) was obtained for all participating sites.

### Health economic methodology

This economic evaluation compared the within-trial costs and consequences of adding enzalutamide to testosterone suppression vs conventional NSAA, in the first 60 months following randomization, using observed individual participant data from the ENZAMET trial, between March 2014 and January 2022. A health economic analysis plan was developed. Costs and health consequences were separated into (i) on-study treatment (i.e. no evidence of disease progression) and (ii) off-study treatment (evidence of disease progression or other reasons for coming off study treatment such as an adverse event, or preference of clinician and patient).

Although enzalutamide was subsidized by the Australian Pharmaceutical Benefits Scheme (PBS), its actual cost was confidential at the time of this analysis. Available sources indicate the price of enzalutamide varies considerably across countries (see Appendix 1 in Supplementary Material). In the US, the price of a single 40 mg tablet ranges from USD$65.91 to USD $130.^15^ Outside the US, prices are generally lower but show an even wider range, from as low as around USD$2 per tablet for generic versions in developing countries to USD$44.58 per tablet in Denmark.^15^^,^^16^ In this analysis, we used the ENZAMET clinical and healthcare utilization data to quantify the volume of the study drugs (enzalutamide and conventional NSAAs) in the base-case analysis, with the aim of estimating the ICER and identifying the price at which enzalutamide would meet the AUD $50,000 per QALY gained willingness-to-pay threshold.

Data for included participants were analyzed on an intention-to-treat basis. Electronic case report forms (eCRF) and participant questionnaires were administered to prospectively collect information on participants’ study status, hospitalizations (including days in an intensive care unit (ICU), days in a high dependency unit (HDU), days in a hospital general ward, presentations to the emergency department (ED)); and health-related quality of life (measured via the EuroQol EQ-5D-5L) at baseline, day 29, week 12, and then every 12 weeks from randomization until clinical progression.

Medicare is the publicly funded universal healthcare insurance scheme in Australia for Australian citizens, permanent residents and eligible visitors. The Australian Medicare Benefits Schedule (MBS) subsidizes the cost of non-drug health services such as diagnostic tests and doctor's visits outside the public hospital system; and the PBS subsidizes the cost of prescription medicines, making them more affordable to individuals. We linked Medicare claims to trial records for consenting Australian participants to assess healthcare use in primary care settings.

This cost-utility analysis was reported according to the Consolidated Health Economic Evaluation Reporting Standards (CHEERS) 2022 checklist.^17^ Details of study data included this economic evaluation analysis are summarized in Table 1. All statistical analyses were performed using SAS (version 9.4).*2.3.*

**Table 1:** Summary of study data prospectively collected in ENZAMET.

Broad item	Description	Included participants	Data source	Period covered
Health outcomes	Overall survival	All randomized participants (n = 1125)	eCRF	Randomization to death (or 19 Jan 2022)
	Progression-free survival	All randomized participants	eCRF	Randomization to clinical progression (or 19 Jan 2022)
	Health-related quality of life (HRQoL)	1105 participants with HRQoL data	Questionnaire survey (paper based & electronically)	Baseline, 29 days, 12 weeks then every 12 weeks until end of study treatment
Healthcare use	Hospitalizations (ED presentations, ICU, HDU, and general ward stays)	All randomized participants	eCRF	29 days, 12 weeks then every 12 weeks until end of study treatment
	Use of health professional services in primary care settings	504 Australian participants with MBS data linkage consent	Medicare MBS claims, Services Australia	From data linkage consent date to death (or 19 Jan 2022)
	Use of prescription medicines in primary care settings	500 Australian participants with PBS data linkage consent	Medicare PBS claims, Services Australia	From data linkage consent date to death (or 19 Jan 2022)

Abbreviations: ED, emergency department; eCRF, electronic case report form; HDU, high dependency unit; HRQoL, health-related quality of life; ICU, intensive care unit.

### Health outcomes

#### Life years

Participants’ disease and survival status were extracted from the trial database. Progression-free and overall survival in the first 60 months were quantified using the Kaplan–Meier method, as per the primary analysis of ENZAMET. Cumulative life years within 60 months of follow-up were estimated by the area under the progression-free and overall survival curve.

#### Quality-adjusted life years

Participants' quality of life whilst on study treatment was assessed with the EuroQoL EQ-5D-5L and EORTC QLQ-C30 instruments. In the base case analysis, participants’ EQ-5D-5L scores were converted into a health utility index, ranging from 0 (death) to 1 (perfect health), using a scoring algorithm developed and validated for Australian adults^18^; these utility scores were then used to calculate quality of life adjustments for surviving participants. Since quality-of-life data following clinical progression or treatment discontinuation (due to adverse effects or preference) were not systematically collected in ENZAMET, off-treatment participants were assigned a health utility value of 0.723. This value was sourced from the post-progression population in the phase-3 ARCHES trial (NCT02677896).^14^

The utility weight for progressive disease was derived from post-progression EQ-5D-5L data, which were mapped to UK EQ-5D-3L preference weights to ensure valuation consistency. Based on the PICO criteria for both ARCHES and ENZAMET, the trial populations were considered comparable, as both enrolled patients with metastatic hormone-sensitive prostate cancer (mHSPC).^14^

Progression-free life years (PFLY) in the first 60 months, defined as the time from randomization to the first progression event or death from any cause, were estimated by the area under the progression-free survival curve out to 60 months of follow-up. Next, life years lived with progressive disease (PLY) were calculated as the difference between life years and progression-free life years. QALYs were estimated by multiplying the mean health utility between data collection time-points for each participant, with the corresponding life-years accumulated in the trial, viz:

$$QALY=\sum_{t=0}\left[\frac{\left(Q_{t}+Q_{t+1}\right)}{2}x\;{PFLY}_{\;t\;to\;t+1}+Q_{progress}x{PLY}_{\;t\;to\;t+1}\right]$$

where Q_t_ refers to the mean utility value obtained in the tth measurement; PFLY refers to progression-free life-years accumulated during the interval between t and t+1; Q _Progress_ refers to the mean utility index for participants with progressive disease; PLY refers to life years lived with progressive disease.

### Healthcare use and costs

#### Study treatment

As per the ENZAMET trial protocol, participants were treated with either enzalutamide 160 mg daily or a standard dose of an older generation NSAA drug (e.g. bicalutamide 50 mg per day) until disease progression or prohibitive toxicity.^7^ PBS pricing was used for unit costs of enzalutamide and NSAA, and the cost of study drugs was multiplied by actual usage as observed in the ENZAMET trial.

#### Other healthcare use

Healthcare use and costs in the first 60 months were identified, measured, and valued from trial records and linked administrative data. Missing data in resource utilization study records (ICU days, HDU days, general ward days, and ED visits) were handled via multiple imputation (MI) using the Multiple Imputation by Chained Equations (MICE) method with predictive mean matching (n = 20 imputations). The imputation model, stratified by allocation, incorporated all longitudinal resource use variables and treatment duration. Data were transposed to wide format prior to imputation to preserve within-patient correlation across 23 study visits, then re-transposed for analysis. Details described in Appendix 2 and Table S1 in Supplementary Material.

The use of observed healthcare resource utilization data enabled the capture of costs associated with treatment-related toxicity in a real-world setting. Costs incurred during each time period were estimated by multiplying the per participant healthcare cost with the corresponding number of active trial participants at each time point. The financial burden of healthcare use during study treatment was evaluated by calculating patient-time exposed to study treatment (from randomization to end of study treatment), which was used to calculate healthcare costs per year of study treatment.

#### Unit costs

Sources for unit costs are outlined in Table 2. Per diem rates of intensive care unit (ICU) and high dependency unit (HDU) stays were taken from the 2020 to 21 National Weight Activity Unit (NWAU) formula developed by the Australian Independent Health and Aged Care Pricing Authority (formerly Independent Hospital Pricing Authority). The per diem rate of general ward stay was obtained from the National Hospital Cost Data Collection (Round 24). Finally, the cost for each MBS or PBS item at current prices were extracted from consenting participants by Services Australia. All costs were adjusted to 2022/2023 (2023) Australian dollars using published deflators (i.e. total health price index) by the Australian Institute of Health and Welfare (AIHW).^19^ Any costs incurred and health outcomes generated 12 months after randomization were discounted at 5.0% per annum as recommended by the Australian Government.^20^ No discounting was applied to costs and outcomes during the first 12 months.

**Table 2:** Unit costs of healthcare resources.

Type of healthcare	Description	Unit	Unit costs (AUD$, 2023)	Source
Study treatment	40 mg Enzalutamide	per tablet	$33^a^	PBS^j^
	50 mg Bicalutamide	per tablet	$2^b^	PBS^j^
Hospitalizations	Intensive care unit (ICU)	per day	$6553^c^	IHACPA^h^
	High dependency unit (HDU)	per day	$6553^d^	IHACPA^h^
	General ward	per day	$2476^e^	NHCDC^e^
	Emergency department (ED)	per presentation	$650^f^	IHACPA^h^
Health services in primary care settings		per MBS claim	Benefits paid^g^	MBS^i^
Prescription medicines in primary care settings		per PBS claim	Benefits paid^g^	PBS^j^

a ENZA = 160 mg oral enzalutamide–4 tablets per day; Enzalutamide 40 mg tablet; The PBS dispensed price for 112 tablets was $3536.96 in 2022 (PBS item 10174L). The price was adjusted to $3684.33 for 2023 using the AIHW Total Health Price Index.

b NSAA = 50 mg oral bicalutamide daily—1 tablet per day; Bicalutamide 50 mg tablet; The PBS dispensed price for 28 tablets was $57.88 in 2022 (PBS item: 8094B). The price was adjusted to $60.29 for 2023 using the AIHW Total Health Price Index.

c Per diem of ICU stay was estimated based on the NWAU 22 calculator for acute activity. According to the NWAU calculator, the hourly price weight of ICU stay was 0.0471 and the National Efficient Price was AUD$5797.

d There is no reported unit cost of HDU stay, assumes the per diem rate of HDU stay equals to the per diem rate of ICU.

e The 2022 national average cost per day in admitted acute was reported in Appendix Table S4. National Hospital Cost Data Collection (NHCDC), Public Sector, Round 24; the 2023 unit cost was calculated using the AIHW total price index.

f Unit cost was calculated by multiplying the price weight for emergency department patients (AECC V1.0: E1290B–Male genitourinary disorders Complexity level B, 0.1122) and the national efficient price at AUD$5797.

g MBS/PBS cost adjusted to 2022/23 $AUD using AIHW total health price index.

h Independent Health and Aged Care Pricing Authority.

i Medical Benefits Schedule.

j Pharmaceutical Benefits Scheme.

### Statistical analysis

As specified in the protocol Schedule of Assessments, trial data on hospital service utilization, including ICU stays, HDU stays, general hospital ward stays, and ED presentations, were collected during the first 29 days post randomization, at 12 weeks and then every 12 weeks thereafter until end of study treatment. The following methodology was used in this analysis to compare the utilization of these healthcare services:

1. Estimate the average volume of healthcare use per patient at each follow-up visit t, denoted as X_t_, along with its corresponding variance, denoted as $S_{t}^{2}$.
2. Estimate the average number of participants on study treatment at each follow-up visit, denoted as P_t_.
3. Calculate the average healthcare use at each visit t by multiplying: X_t_ P_t_.
4. Calculate the corresponding variance at visit *t* as: $S_{t}^{2}$ $P_{t}^{2}$.
5. Estimate the total average volume of healthcare use from randomization to 60 months by summing the estimates from visits 1 to 23.
6. Assuming no statistical correlation between visits, the overall variance was calculated as the sum of the visit-level variances from visit 1 to visit 23.
7. Per patient-year healthcare use was calculated by dividing the total average healthcare use by the total time of exposure for on-treatment participants (denoted as *P*).
8. The variance of per patient-year healthcare use was calculated by dividing the total variance by P^2^.
9. Difference in per patient-year healthcare use between treatment arms was examined using t statistics assuming equal means. The t statistics were calculated using the standard formulas below:

$$t=\frac{{\bar{X}}_{1}-{\bar{X}}_{2}}{s_{p}\;\sqrt{\frac{1}{n_{1}}+\frac{1}{n_{2}}}}$$

where,

$$s_{p}=\sqrt{\frac{{\left(n_{1}-1\right)s}_{X_{1}}^{2}+{\left(n_{2}-1\right)s}_{X_{2}}^{2}}{n_{1}+n_{2}-2}}$$

This method was only applied to healthcare use data collected from CRFs (i.e., hospitalization data). It did not apply to linked Medicare data, which covered around 45% of ENZAMET trial participants that consented to data linkage (504 for MBS and 500 for PBS).

### Base case analysis

Within-trial cost-effectiveness of adding enzalutamide and conventional NSAA to first-line therapy of mHSPC was analyzed by calculating the ICER, by dividing the incremental cost of enzalutamide management vs usual care by the corresponding incremental QALYs, using best practice methods. The ICER represents the average additional cost associated with gaining 1 additional life-year or QALY. An implicit willingness-to-pay threshold of AUD$50,000 per QALY gained was used as the criterion of good value for money.^21^

### Sensitivity analyses

#### One-way sensitivity analysis

One-way sensitivity analyses were undertaken to check the robustness of the results, using a range of upper and lower estimates from ENZAMET and other sources. These included varying the unit price of study drugs, per diem rates of hospital stays, the benefits paid from MBS and PBS claims; and the quality-of-life scores underlying health utilities. For the cost of enzalutamide, a wide range of plausible unit prices was applied, varying from a minimum of AUD $3 to a maximum of AUD $196; for other cost inputs, standard multipliers of 0.5–2.0 were applied. In addition, cost-effectiveness analyses were conducted with the discount rate set from 5% in the base case to 0% (i.e. undiscounted). Health utilities for on-treatment participants were varied using a second set of recently published EQ-5D-5L value sets for Australians^22^ and the EORTC QLU-C10D,^23^ which is an Australian validated cancer-specific utility instrument derived from the EORTC QLQ-C30. For off-treatment participants, two alternative health utility values (0.688 and 0.706), as reported in the Enzalutamide PBAC Public Summary Document (March 2023),^14^ were applied in the one-way sensitivity analysis. The first utility value was derived from baseline EQ-5D-3L data from the AFFIRM trial (NCT00974311) and converted using UK preference weights, while the second value represented the average utility across the ARCHES trial (base case) and the AFFIRM trial.^14^

#### Probabilistic sensitivity analysis (PSA)

For the PSA, a Monte Carlo simulation with 100,000 replications was conducted using the assigned distributions for cost and health utility variables to assess parameter uncertainty. Based on the 2012 ISPOR-SMDM report, beta (β) distributions were applied to health utility parameters, while gamma (γ) distributions were applied to all cost parameters.^24^ Precision estimates around the mean included standard deviations, standard errors, and upper and lower 95% confidence intervals. Details of the PSA are reported in Appendix 4 & Table S5.1 in Supplementary Material. A cost-effectiveness acceptability curve (CEAC) was generated to show the probability of enzalutamide being cost effective at WTP thresholds of AUD$20,000–$100,000 per QALY gained.

### Role of the funding source

The funder of the study had no role in study design, data collection, data analysis, data interpretation, or writing of the report.

## Results

### Health outcomes

#### Base case overall and progression-free survival

In the first 60 months following randomization, participants randomized to enzalutamide accrued a mean of 4.25 life-years (i.e. overall survival), which was 0.27 years greater than those randomized to NSAA, mean of 3.98 life-years (see Table 3). For progression-free survival, the enzalutamide arm accrued a mean of 3.78 progression-free life-years, 1.12 years more than the NSAA arm, which accrued 2.66 years.

**Table 3:** Health outcomes observed at 60 months.

Health outcomes	Undiscounted		Discounted	
	Enzalutamide	NSAA	Enzalutamide	NSAA
Life years	4.25	3.98	3.90	3.66
Progression-free life years	3.78	2.66	3.47	2.49
Quality-adjusted progression-free life years^a^	2.95	2.17	2.71	2.02
Quality-adjusted life years (QALYs)^a^^,^^b^	3.29	3.11	3.02	2.87

Abbreviations: NSAA, non-steroidal ant-androgen; QALY, quality-adjusted life year.

a Health utilities for on-treatment participants were derived using the 2013 Australian value set for the EQ-5D-5L (Norman et al., 2013) in the base case, resulting in mean utility values of 0.78 for enzalutamide and 0.81 for NSAA. No marked differences were observed when participants' quality of life was assessed and valued using the cancer-specific EORTC QLU-C10D (King et al., 2018). In sensitivity analyses applying the 2023 Australian EQ-5D-5L value set (Norman et al., 2023), the mean health utilities for on-treatment participants increased to 0.90 for enzalutamide and 0.92 for NSAA.

b Health utility for off-treatment participants was assumed to be 0.723.

##### Health-related quality of life (HRQoL)

A total of 1105 (98%) participants–553 (98%) in the enzalutamide group and 552 (98%) in the NSAA group–completed 15,332 EQ-5D-5L assessments. Of these, 14,870 (97%) assessments were completed while participants were on study treatment (i.e. progression-free), with 8751 from the enzalutamide group and 6119 from the NSAA group. Fig. 1 shows the mean EQ-5D-5L health utilities by treatment group and study treatment status. Among on-treatment participants, the mean EQ-5D-5L utility score was 0.783 for those randomized to enzalutamide, which was 0.027 lower (p < 0.0001) than the utility for those randomized to NSAA at 0.810. However, this difference in health utility did not exceed the commonly used minimally important clinical difference of 0.03 for Australian cancer patients.^23^

**Fig. 1:**
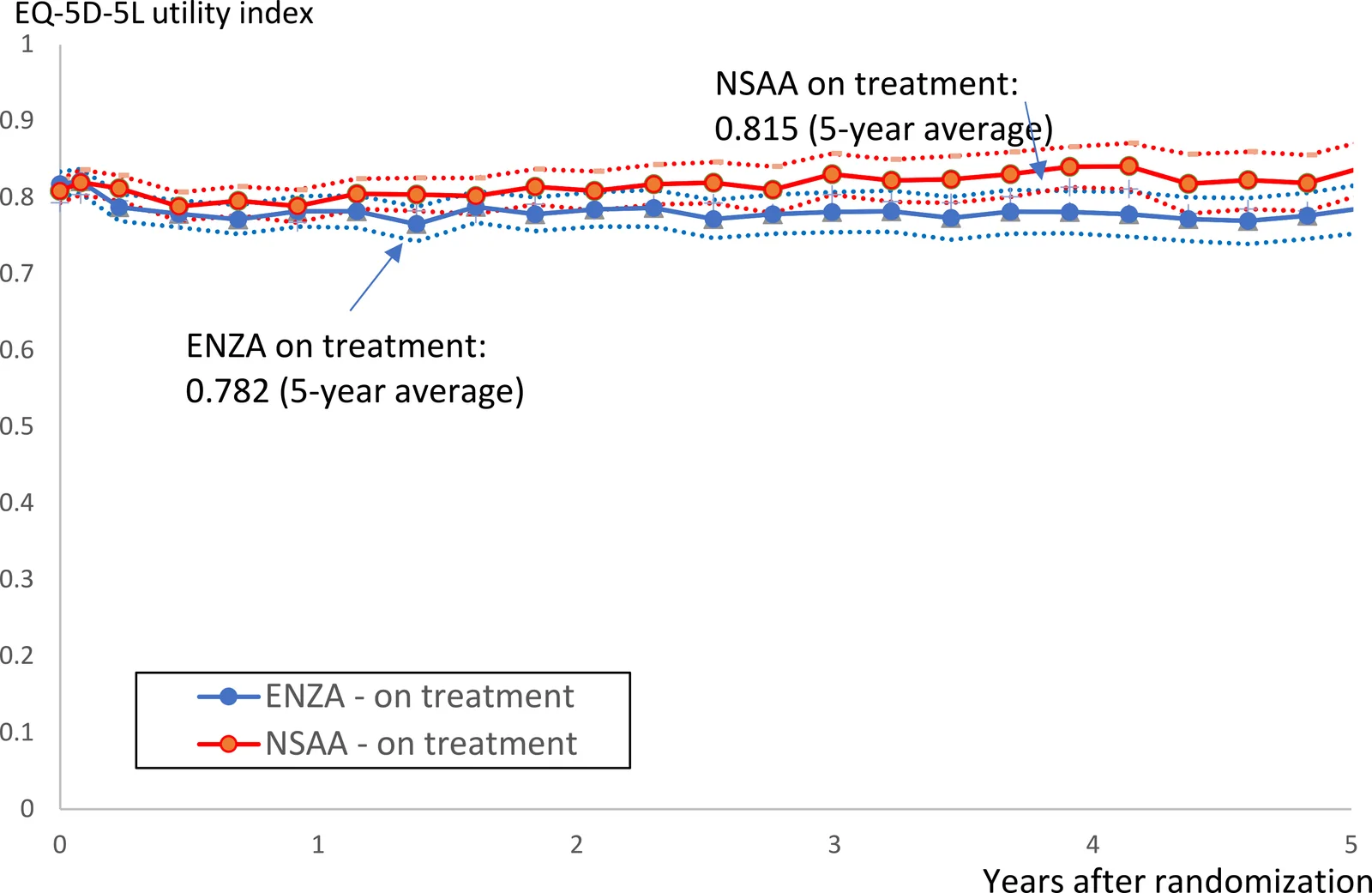
Average health utilities using Australian EQ-5D-5L value sets. Abbreviations: ENZA, enzalutamide; NSAA, non-steroidal antiandrogen. Footnote: Participants' EQ-5D-5L profile was converted into utility index using the 2013 value set (Norman et al., 2013).

##### Base case quality-adjusted life years

Table 3 shows the 60-month quality-adjusted survival and quality-adjusted progression-free survival by treatment group. Incorporating EQ-5D-5L data from the ENZAMET trial, the enzalutamide group was estimated to have accrued 2.95 quality-adjusted progression-free life years, compared with 2.17 years for the NSAA group. Further analysis showed the enzalutamide group accrued 3.29 quality-adjusted life years (QALYs) over 60 months, while the NSAA group accrued 3.11 QALYs. After applying discounting, the enzalutamide group accrued 3.02 QALYs, which was 0.15 more than the NSAA group (2.87 QALYs).

### Base case healthcare use and costs

In the first 60 months following randomization, trial participants randomized to enzalutamide accrued 724,298 person-days on study treatment, which was about 47% more than those to NSAA at 493,230 days. This reflects the longer time on treatment due to higher efficacy of enzalutamide and lower rates of cessation of treatment due to progression or toxicity. After incorporating trial data on participant's dose modification, it was estimated 2,780,000 enzalutamide tablets and 493,000 NSAA tablets were dispensed for study treatment during the trial. (see Appendix 3 in Supplementary Material for details). Based on PBS-listed prices, this corresponded to an estimated monetary value of $84.3 million for enzalutamide and $997 thousand for NSAA drugs over the course of the trial.

In addition to the intervention medicines, healthcare use including hospitalizations and primary healthcare for ENZAMET participants in the first 60 months were presented in Table 4.

**Table 4:** Healthcare use and costs, base case.

	Enzalutamide group (n = 563)					NSAA group (n = 562)				
	Healthcare use^d^		Cost (AUD$, 2023)			Healthcare use^d^		Cost (AUD$, 2023)		
	Per patient-year^b^	Per patient	Per patient-year^b^	Per patient, undiscounted	Per patient, discounted	Per patient-year^b^	Per patient	Per patient-year^b^	Per patient, undiscounted	Per patient, discounted
On study treatment										
Hospitalizations										
ICU^a^	0.05	0.17	$311	$1106	$1008	0.02	0.04	$111	$269	$253
HDU^a^	0.02	0.06	$104	$369	$343	0.01	0.02	$51	$124	$116
General ward^a^	1.12	3.99	$2777	$9869	$9278	0.92	2.23	$2277	$5518	$5264
ED^a^	0.21	0.75	$138	$490	$455	0.21	0.51	$137	$331	$319
Subtotal				$11,834	$11,084				$6242	$5952
Medicare claims^c^										
MBS^a^^,^^c^	60	214	$3392	$12,054	$11,196	65	157	$3883	$9411	$8891
PBS^a^^,^^c^	45	160	$6310	$22,426	$20,830	52	125	$7677	$18,604	$17,619
Subtotal				$34,480	$32,026				$28,015	$26,510
Overall hospitalizations and Medicare claims				$46,314	$43,110				$34,257	$32,462
Off study treatment										
Medicare claims^c^										
MBS^a^^,^^c^	104	72	$8296	$5791	$5151	92	138	$7051	$10,608	$9461
PBS^a^^,^^c^	61	43	$18,700	$13,054	$11,710	62	93	$29,262	$44,101	$39,393
Overall Medicare claims				$18,845	$16,861				$54,709	$48,854
All healthcare use from randomization to 60 months (excluding study drugs)				$65,159	$59,971				$88,966	$81,316

Abbreviations: AUD, Australian dollar; ED, emergency department; HDU, high dependency unit; ICU, intensive care unit; MBS, Medicare Benefits Schedule; PBS, Pharmaceuticals Benefits Scheme.

a Unit of resource use: Days in Intensive Care Unit (ICU); Days in High Dependency Unit (HDU); Days in General ward; Number of Emergency Department (ED) presentations; Number of MBS claims; Number of PBS claims.

b Patient-years: Enzalutamide on treatment–2001; NSAA on treatment–1362; Enzalutamide off treatment–393; NSAA off treatment—847.

c Linked administrative (Medicare) data only available for trial participants resident in Australia.

d The purpose of Table 4 is to illustrate the overall healthcare utilisation and costs for ENZAMET participants by treatment allocation. Therefore, only the mean values of healthcare utilisation and costs are reported in Table 4. Including additional information would affect the readability of the table. The corresponding p-values are reported in Tables S2.1, S2.2, S2.3 and S2.4 in the Supplementary Material.

#### Hospitalizations (on study treatment only)

The enzalutamide group incurred a longer duration of hospital stays than the NSAA group (see Table 4). When adjusted for patient-years exposed to study treatment, participants randomized to receive enzalutamide incurred slightly more time in intensive care (Enzalutamide = 0.047 days vs NSAA = 0.017, p = 0.01 days; Table S2.1 in Supplementary Material) and general wards (Enzalutamide = 1.121 days vs NSAA = 0.920 days, p = 0.03; Table S2.3 in Supplementary Material) than those randomized to NSAA. No significant differences in per patient-year high dependency unit (HDU) stays (Enzalutamide = 0.016 days vs NSAA = 0.008 days, p = 0.25; Table S2.2 in Supplementary Material) or emergency department (ED) presentations (Enzalutamide = 0.210 vs NSAA = 0.210, p = 0.99; Table S2.4 in Supplementary Material) were observed between randomized groups.

#### Use of medical services and prescription medicines in the primary care setting

Linked administrative data were extracted from Australian trial participants (n = 504 [45% of trial population] for MBS and n = 500 [44%] for PBS). Analyses were conducted separately for periods during study treatment and after study treatment (see Table 4).

For on-treatment participants, those in the enzalutamide group incurred slightly lower mean MBS claims per patient-year (60 vs 65) and lower mean MBS costs per patient-year (AUD$3400 vs $3900) compared those in the NSAA group. However, due to longer progression-free survival and extended exposure to study treatment, enzalutamide participants accrued higher overall claims (MBS: 214 vs 157; PBS: 160 vs 125) and costs (MBS: AUD$11,200 vs AUD$8900; PBS: AUD$20,800 vs AUD$17,600) than NSAA participants.

Among off-treatment participants (see Table 4), the enzalutamide group incurred more MBS claims per patient-year compared to the NSAA group (104 vs 92), while PBS claims for non-study drugs per patient-year were similar between groups (61 vs 62). Despite having longer progression-free survival, the enzalutamide group experienced a shorter duration of progressive disease in the off-treatment phase. As a result, the enzalutamide arm incurred fewer claims per patient (MBS: 72 vs 138; PBS: 43 vs 93) and lower costs per patient (MBS: $5200 vs $9500; PBS: $11,700 vs $39,400), compared with the NSAA group.

##### MBS claims and costs by broad type of service (BTOS) classification

Approximately 50% (n = 54,720 [out of 108,250]) of MBS claims for all participants during study treatment were for pathology services, followed by professional attendances (∼30%; n = 35,641), therapeutic procedures (∼7%; n = 7828), and diagnostic imaging services (∼5%; n = 5382) (see Fig. 2 or Table S3.1 in Supplementary Material). For participants who were off treatment, the proportion of MBS claims for therapeutic procedures was slightly higher than those on treatment (10% [6496 out of 59,943] vs 7% [n = 7828]).

**Fig. 2:**
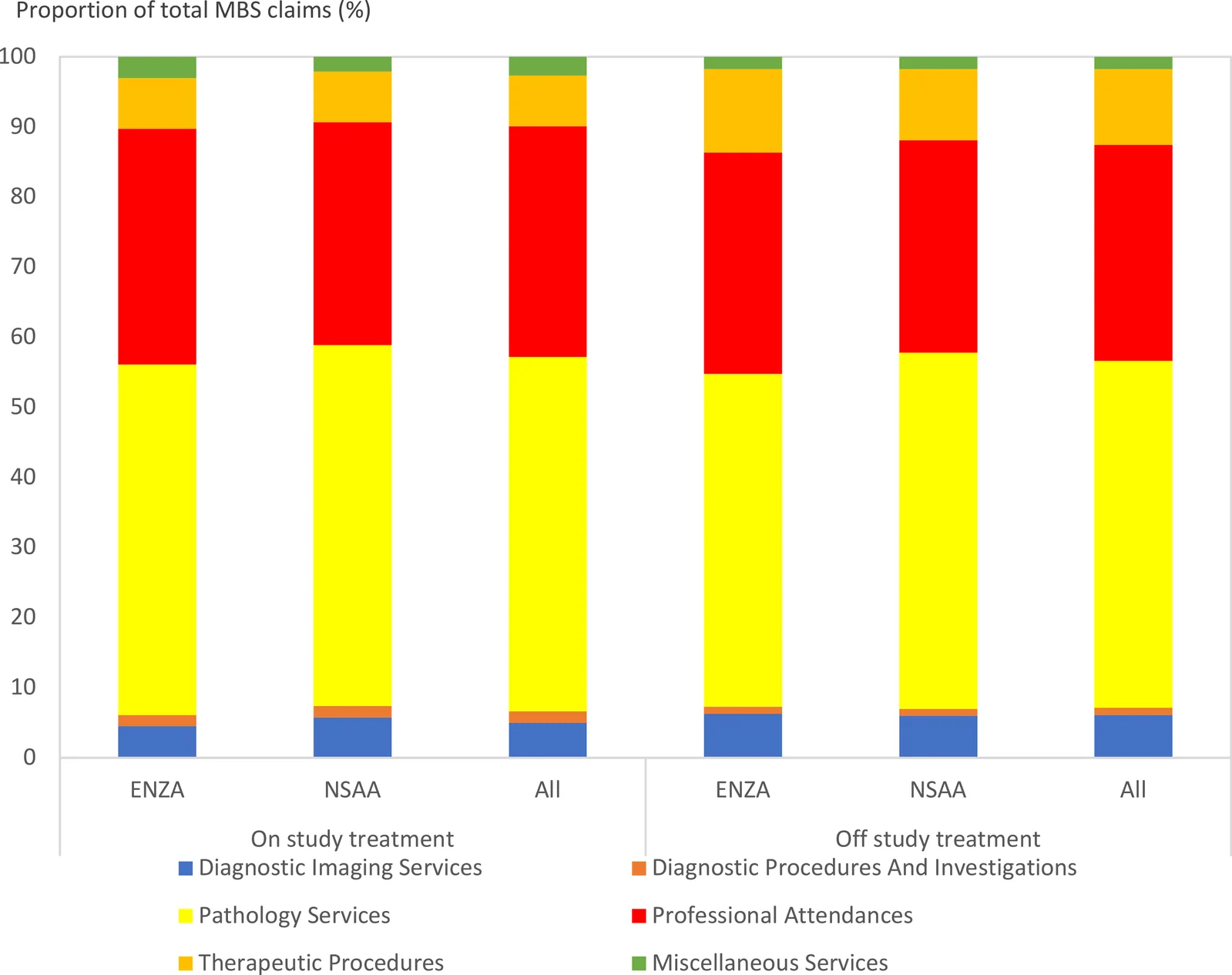
Distribution of MBS claims by Broad Type of Service (BTOS) classification. Abbreviations: BTOS, Broad Type of Service; ENZA, enzalutamide; MBS, Medicare Benefits Schedule; NSAA, non-steroidal antiandrogen.

For MBS costs among on-treatment participants (see Fig. 3 or Table S3.2 in Supplementary Material), the largest proportion was attributed to professional attendances (37%; AUD$2.3 million out of AUD$6.3 million), followed by diagnostic imaging services (23%; AUD$1.4 million), therapeutic procedures (20%; AUD$1.3 million) and pathology services (15%; AUD$950k). When comparing treatment arms, on-treatment participants randomized to enzalutamide had a higher proportion of MBS costs for professional attendance (40%; AUD$1.5 million [out of AUD$3.8 million] vs 34%; AUD$863k [out of AUD$2.5 million]) and a lower proportion for diagnostic imaging services (20%; AUD$759k vs 27%; AUD$677k) compared to those receiving NSAA.

**Fig. 3:**
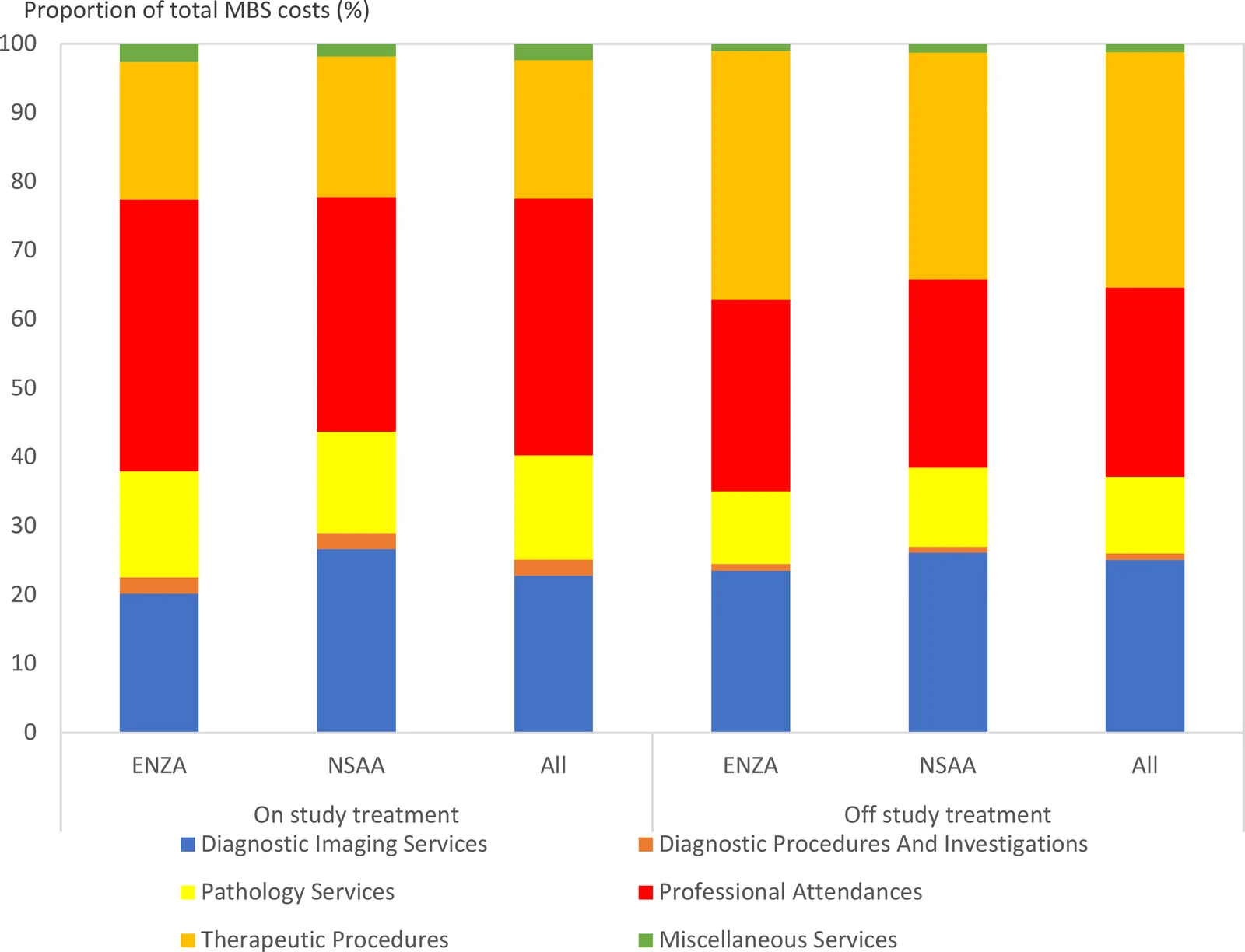
Distribution of MBS costs by broad type of service (BTOS) classification. Abbreviations: BTOS, Broad Type of Service; ENZA, enzalutamide; MBS, Medicare Benefits Schedule; NSAA, non-steroidal antiandrogen.

For off-treatment participants, the proportion of MBS costs for therapeutic procedures was higher (34%; AUD$1.6 million [out of AUD$4.6 million] vs 20%; AUD$1.3 million), and the proportion for professional attendances was lower (28%; AUD$1.3 million vs 37%; AUD$2.3 million) compared with on-treatment participants (see Fig. 3 or Table S3.2 in Supplementary Material). Between treatment arms, off-treatment participants in the enzalutamide group had a slightly higher proportion of MBS costs for therapeutic procedures (36%; $650k [out of AUD$1.8 million] vs 33%; AUD$927k [out of $2.8 million]) and a slightly lower proportion for diagnostic imaging services (24%; AUD$423,000 vs 26%; AUD$736,000) than those in the NSAA group.

##### PBS claims and costs by therapeutic area

Among on-treatment participants, 35% of PBS claims were for cardiovascular medications; followed by antineoplastic and immunomodulating agents (20%; n = 16,410 [out of 82,289]); alimentary tract and metabolism (14%; n = 11,777); and nervous system (12%; n = 10,069), based on the World Health Organization's Anatomical Therapeutic Chemical (ATC) Classification (see Fig. 4 or Table S4.1 in Supplementary Material). When comparing treatment arms, on-treatment enzalutamide participants incurred a higher proportion of PBS claims for cardiovascular (39%; n = 18,906 [out of 49,052] vs 29%; n = 9582 [out of 33,237]), musculoskeletal (5%; n = 2442 vs 3%; n = 872), nervous system (14%; n = 7022 vs 9%; n = 3047) and alimentary tract and metabolism agents (16%; n = 7811 vs 12%; n = 3966). However, they incurred a lower proportion of PBS claims for antineoplastic & immunomodulating agents (11%; n = 5389 vs 33%; n = 11,021) compared with NSAA participants.

**Fig. 4:**
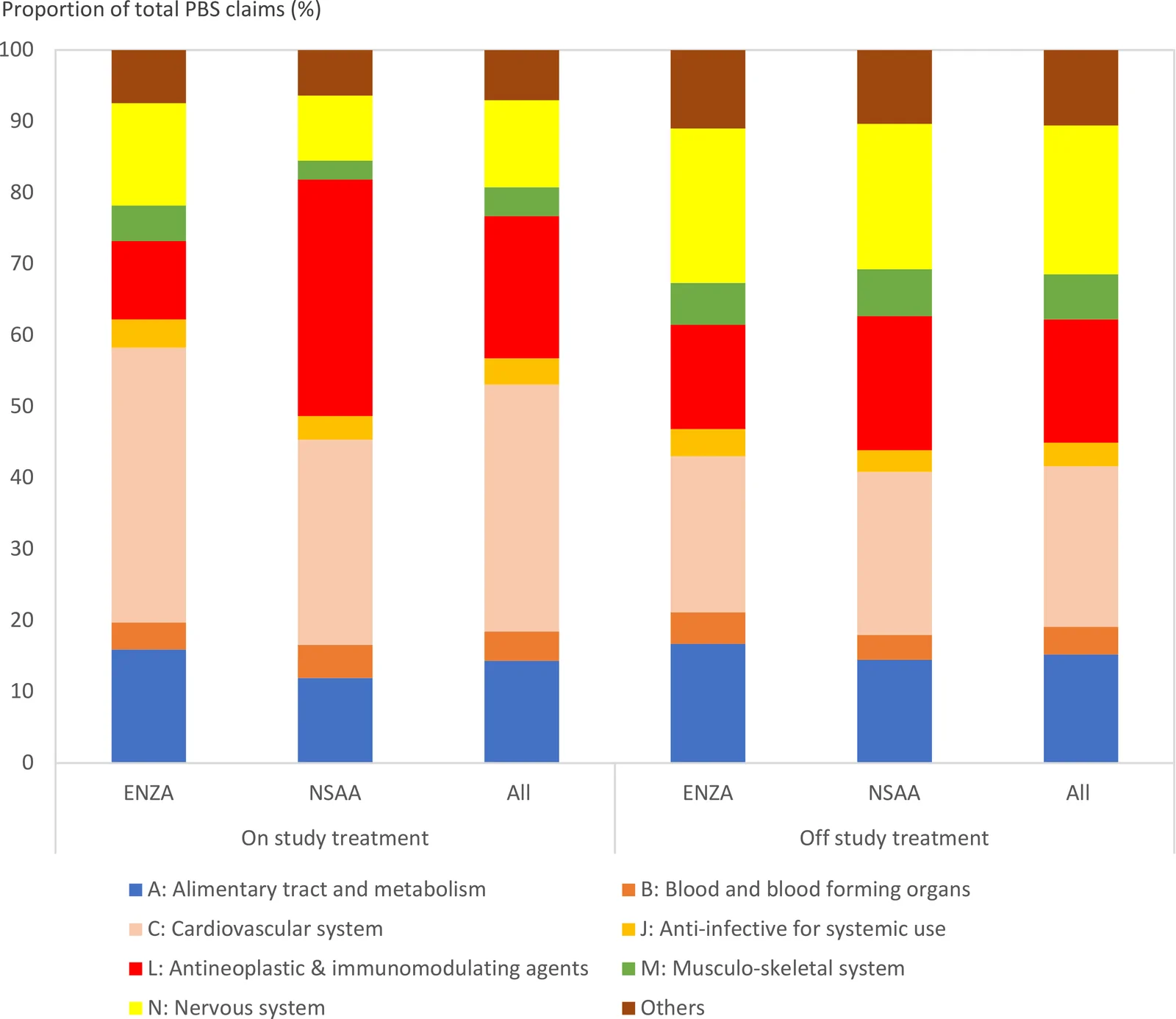
Distribution of PBS claims by anatomic therapeutic chemical classification (1st level). Abbreviations: ENZA, enzalutamide; PBS, Pharmaceuticals Benefits Scheme; NSAA, non-steroidal antiandrogen.

For off-treatment participants, the distribution of PBS claims was broadly similar, though there were fewer claims for cardiovascular treatments (23%; n = 8502 [out of 37,710] vs 35%; n = 28,488 [out of 82,289]) and more for nervous system medications (21%; n = 7860 vs 12%; n = 10,069). (see Fig. 4 or Table S4.1 in Supplementary Material). In the off-treatment group, enzalutamide participants incurred a higher proportion of PBS claims for alimentary tract and metabolism medications (17%; n = 2229 [out of 13,308] vs 14%; n = 3524 [out of 24,402]) and a lower proportion of PBS claims for antineoplastic & immunomodulating agents (15%; n = 1943 vs 19%; n = 4586) when compared with NSAA participants.

Antineoplastic and immunomodulating medications were the primary contributors to PBS costs, accounting for 81% of costs (AUD$95 million out of AUD$11.7 million) in on-treatment participants and 90% (AUD$13.3 million out of AUD$14.9 million) in off-treatment participants (see Fig. 5 or Table S4.2 in Supplementary Material). Musculoskeletal medications were the second-highest contributors, representing 4% of PBS costs (AUD$470k) for on-treatment participants and 6% (AUD$827k) for off-treatment participants. Prescribed cardiovascular medicines accounted for a relatively small proportion of PBS costs–3% for on-treatment (AUD$338k) and 1% for off-treatment (AUD$126k).

**Fig. 5:**
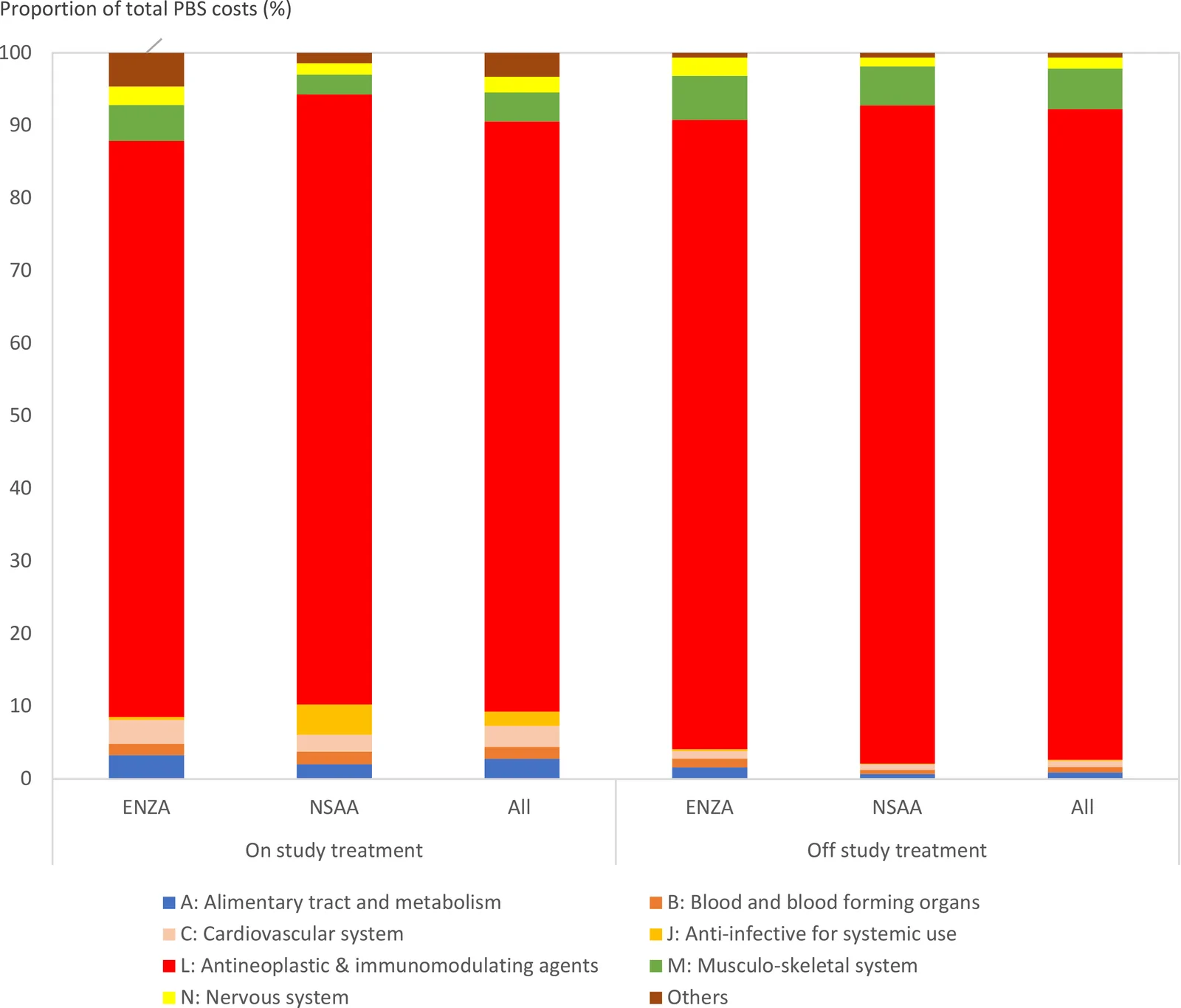
Distribution of PBS costs by anatomic therapeutic chemical classification (1st level). Abbreviations: ENZA, enzalutamide; PBS, Pharmaceuticals Benefits Scheme; NSAA, non-steroidal antiandrogen.

### Base case analysis

Results for the base-case cost-effectiveness analyses for enzalutamide and NSAA are shown in Table 5. In the base case, the enzalutamide group with a PBS-listed price of AUD$33 per tablet, resulted in an additional AUD$127,262 per patient costs, 0.24 life-years gained, and 0.15 QALYs gained in the first 5 years, compared with the NSAA group. This equated to an ICER of AUD$543,880 per life-year saved and AUD$835,580 per QALY gained. The cost of the study drugs was the primary driver of incremental costs, accounting for more than 100% of the total (AUD$148,607), a figure which indicates cost offsets in other areas for the enzalutamide group. When enzalutamide was priced at AUD$6.72 per tablet (i.e. 20.4% of the current PBS-listed price), the estimated ICER met the willingness-to-pay threshold of AUD $50,000 per QALY gained.

**Table 5:** Base-case incremental cost-effectiveness analysis.

	Undiscounted	Discounted (5% per annum)
Incremental cost, per patient (AUD)		
Study drugs	$161,100	$148,607
Hospitalizations		
ICU	$837	$755
HDU	$245	$227
General ward	$4351	$4014
ED	$159	$136
MBS		
On treatment	$2643	$2305
Off treatment	-$4817	-$4310
PBS		
On treatment	$3822	$3211
Off treatment	-$31,047	-$27,683
Overall	$137,293	$127,262
Incremental effectiveness, per patient		
Life years	0.27	0.24
QALYs	0.18	0.15
Incremental cost-effectiveness ratio (AUD)		
Cost per Life year saved	$508,498	$543,880
Cost per QALY gained	$773,026	$835,580

Abbreviations: AUD, Australian dollar; ED, emergency department; HDU, high dependency unit; ICU, intensive care unit, MBS, Medicare Benefits Schedule; PBS, Pharmaceuticals Benefits Scheme; QALY, quality-adjusted life year.

#### Analysis of healthcare costs (excluding study treatment)

When the study treatment cost was excluded, on-treatment participants randomized to enzalutamide incurred average healthcare costs of AUD $43,100 per patient, which was $10,600 higher than the $32,500 incurred by the NSAA group (Table 4). In contrast, off-treatment participants in the enzalutamide group incurred average costs of AUD $16,900, which was $32,000 lower than the $48,900 for the NSAA group. Consequently, the total average healthcare cost per patient, excluding the study drug, was AUD $60,000 for enzalutamide, ($21,300 lower than the $81,300 for NSAA).

For participants on treatment, prescribed medicines and primary care services were the main cost components. Medicare MBS/PBS claims constituted approximately 74% (AUD$32,000 of $43,200) of costs in the enzalutamide group and 81% (AUD$26,500 of $32,700) in the NSAA group (Table 4).

Regarding hospitalizations, more than 80% of costs were attributable to general ward stays. Notably, per-patient costs for general ward stays were 76% higher among participants randomized to enzalutamide compared with those in the NSAA group.

### Sensitivity analyses

Results of one-way sensitivity analyses are shown in Table 6. Enzalutamide became a dominant strategy, i.e. lower in cost and associated with greater health gains, when its per tablet price was reduced to AUD$3 (i.e. as per generic versions available in LMIC countries). Variations in other cost inputs and health utility values did not materially affect the base-case results.

**Table 6:** One-way sensitivity analyses for cost-effectiveness.

	Incremental cost per life-year saved	Incremental cost per QALY gained
Base case	$543,880	$835,580
*One-way sensitivity analyses*		
1. Per tablet cost of enzalutamide increased to AUD$67 (Denmark)	$1,205,479	$1,852,018
2a. Per tablet cost of enzalutamide decreased to AUD$25	$388,208	$596,417
2b. Per tablet cost of enzalutamide decreased to AUD$20	$290,914	$446,941
2c. Per tablet cost of enzalutamide decreased to AUD$15	$193,620	$297,464
2d. Per tablet cost of enzalutamide decreased to AUD$10	$96,325	$147,988
2e. Per tablet cost of enzalutamide decreased to AUD$3 (Generic version available in developing countries)^a^	Enzalutamide dominates	Enzalutamide dominates
3. Per tablet cost of NSAA drugs increased by 100%	$536,838	$824,762
4. Per tablet cost of NSAA drugs decreased by 50%	$547,399	$840,988
5. General ward stay per diem increased by 100%	$561,033	$861,934
6. General ward stay per diem decreased by 50%	$535,301	$822,402
7. MBS benefits increased by 100%	$535,310	$822,415
8. MBS benefits decreased by 50%	$548,163	$842,161
9. PBS benefits increased by 100%	$439,293	$674,900
10. PBS benefits decreased by 50%	$596,172	$915,919
11. On-treatment health utility valued using EORTC QLU-C10D	$543,880	$919,163
12. On-treatment health utility valued using 2023 EQ-5D-5L value sets	$543,880	$432,160
13. Off-treatment health utility at 0.706	$543,880	$770,847
14. Off-treatment health utility at 0.688	$543,880	$712,409

Abbreviations: AUD, Australian dollar; EORTC, European Organisation for Research and Treatment of Cancer; MBS, Medicare Benefits Schedule; NSAA, non-steroidal anti-androgen; PBS, Pharmaceuticals Benefits Scheme; QALY, quality-adjusted life year.

a In Sensitivity Analysis 2e, which reduced the per-tablet cost of enzalutamide to AUD$3, the within-trial analysis resulted in enzalutamide becoming a dominant strategy, with an estimated incremental cost of -AUD$9333, an incremental gain of 0.24 life-years, and an incremental gain of 0.15 QALYs.

Across 100,000 PSA simulations, the cost-effectiveness scatterplot is presented in Fig. 6. The average costs and QALYs were consistent with the deterministic base-case findings, yielding a mean incremental cost of AUD$126,900 (SD = $74,600) and a mean QALY gain of 0.15 (SD = 0.92) (see Table S.2 in Supplementary Material). Based on the PSA results, enzalutamide had a 6% probability of being cost-effective at a WTP threshold of AUD$50,000 per QALY gained. Further analysis using the cost-effectiveness acceptability curve (Fig. 7) indicated that the probability of enzalutamide being cost-effective increased to 16% when the WTP threshold was increased to AUD$100,000 per QALY gained.

**Fig. 6:**
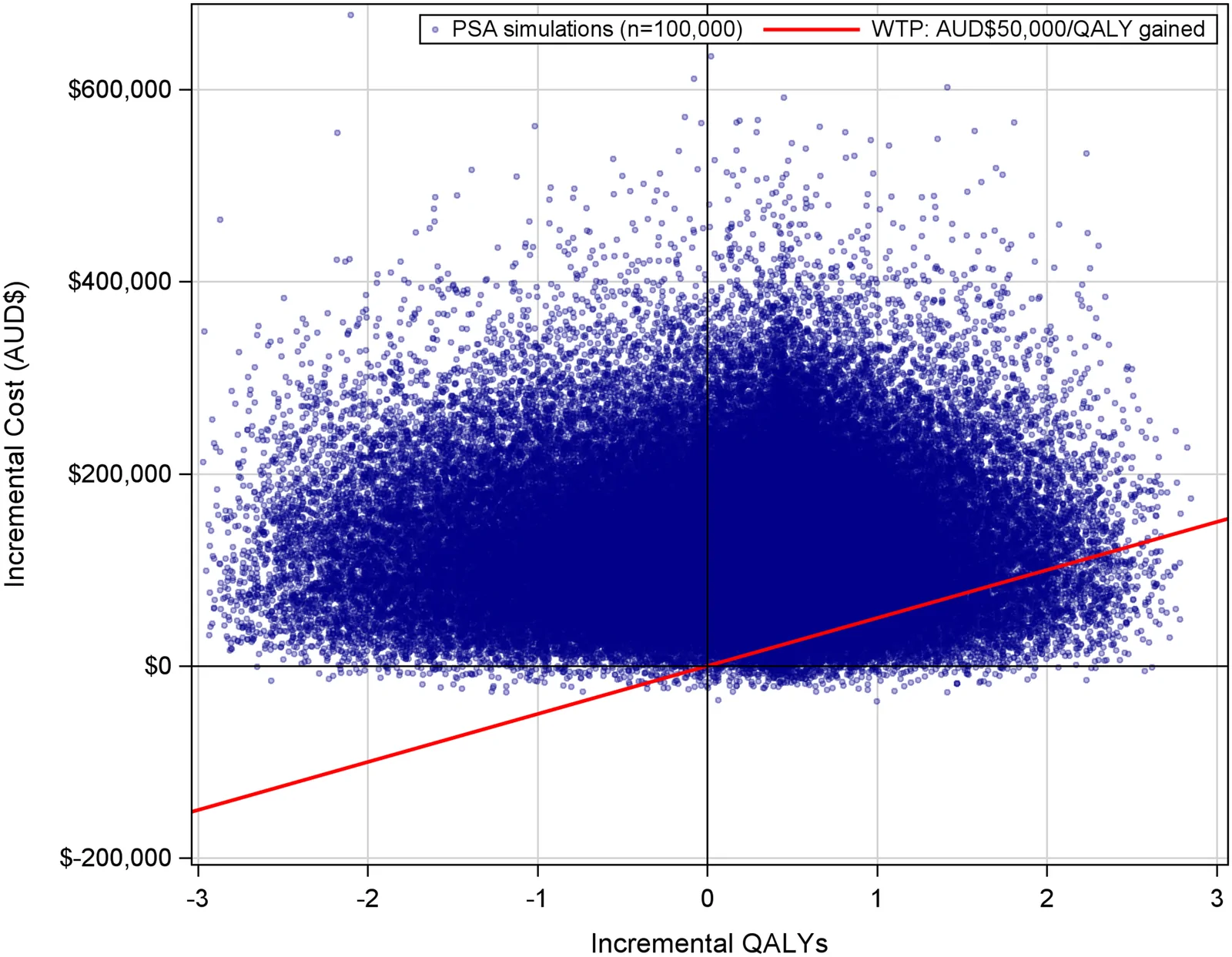
Cost-effectiveness plane–Enzalutamide vs NSAA. Abbreviations: AUD, Australian dollar; NSAA, non-steroidal antiandrogen; PSA, probabilistic sensitivity analysis; QALY, quality-adjusted life years; WTP, willingness-to-pay.

**Fig. 7:**
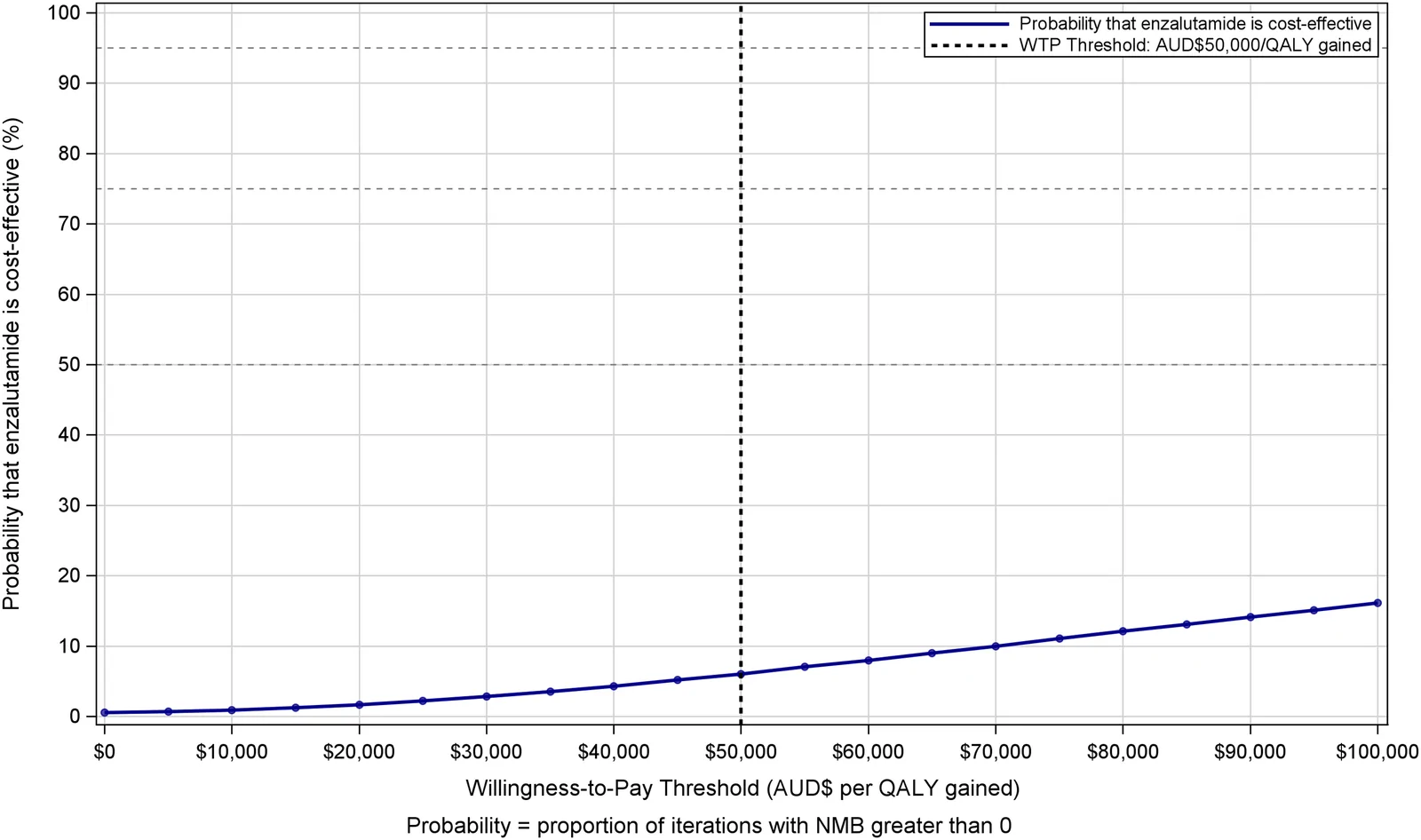
Cost-effectiveness acceptability curve-Enzalutamide vs NSAA. Abbreviations: AUD, Australian dollar; QALY, quality-adjusted life year; NMB, net monetary benefit; NSAA, non-steroidal antiandrogen; WTP, willingness-to-pay.

## Discussion

This within-trial analysis used real-world data observed in the large, international ENZAMET trial to evaluate the cost-effectiveness of adding enzalutamide to testosterone suppression for men with mHSPC. A key strength of the study is the use of observed healthcare resource utilization data, which likely captures costs associated with treatment-related toxicity in a real-world setting, in contrast to existing modeled evaluations.^9^^,^^14^

Despite clinical evidence that showed enzalutamide significantly improved overall and progression-free survival, the additional cost of the study drug at the PBS-listed price of AUD$33 per tablet, to obtain a modest increase in quality-adjusted survival, meant enzalutamide in this context exceeded Australia's generally accepted willingness-to-pay threshold of AUD$50,000 pr QALY. We noted, the PBAC Public Summary Document (March 2023), advised the price of enzalutamide, should not be higher than the price of apalutamide at an equi-effective daily dose. The PBS currently subsidizes apalutamide at AUD$124 per day [PBS website], which is similar to the daily cost of enzalutamide.

Our economic results were consistent with four previous model-based evaluations conducted from the US and Chinese payer perspectives.^9^ In the US, the ICER for addition of enzalutamide to androgen deprivation therapy (ADT) vs ADT alone was US$430,934/QALY gained; and in China it was US$225,445/QALY gained.^9^ Both of these ICERs exceeded commonly used cost-effectiveness thresholds. A third study in the US compared enzalutamide plus ADT with abiraterone plus ADT, generating an ICER of US$509,813 per QALY gained. This is slightly lower but very similar to our result of AUD$835,580 (∼US$580,676, PPP) per QALY gained which was further confirmed within the Australian context through both one-way and probabilistic sensitivity analyses. A final US study reported enzalutamide would become cost-effective when a 47% discount on current pricing was applied.^11^ Furthermore, one-way sensitivity analyses showed the drug could become a dominant strategy at lower pricing, allowing greater flexibility for Government agencies to subsidize it after patent expiry. To be considered good value for money at the implicit willingness-to-pay threshold, the unit price would need to be reduced by around 80% from the current PBS-listed price.

Although enzalutamide has demonstrated clinical benefits in improving progression-free and overall survival for patients with HSPC, its potential adverse effects on bone health warrants careful consideration. This analysis revealed a higher proportion of PBS claims and associated costs for musculoskeletal system medications in the enzalutamide group, which suggests a possible link to increased bone-related complications. To support this, a recent systematic review of eleven randomized trials showed that the use of enzalutamide in patients with prostate cancer has been associated with increased risk of falls and bone fractures.^25^ A similar pattern was observed in the ENZAMET trial, where rates of all-grade falls (4.1 vs 1.8 per 100 patient-years) and all-grade fractures (2.0 vs 1.1 per 100 patient-years) were higher in enzalutamide participants compared to those on NSAA.^8^ These findings highlight the need for further research to evaluate the impact of falls and fractures on patient quality of life, healthcare resource use, and the overall cost of treatment with potent androgen receptor signaling inhibitors such as enzalutamide, apalutamide, darolutamide, and abiraterone. Studies assessing the economic value of preventive strategies for bone health in this population are also needed.

This study has some limitations. First, although ENZAMET was a multicenter randomized controlled trial conducted across six countries, detailed data on participants’ healthcare use in the primary care setting (e.g. diagnostic tests, healthcare visits) were only available for the subgroup of Australian participants who consented to data linkage. While these healthcare utilization patterns may be broadly representative of the overall study cohort, differences in healthcare systems may limit the generalizability of cost estimates beyond the Australian context. Nonetheless, the frequency and types of healthcare events reported in the trial records can inform cost-effectiveness modeling in other jurisdictions, using local healthcare cost structures and drug pricing (on- and off-patent).

Second, participants randomized to ENZAMET may not fully represent the broader population of patients with metastatic hormone-sensitive prostate cancer (mHSPC) treated with enzalutamide in routine clinical practice. Trial participants are generally healthier and less burdened by comorbidities, which may influence both clinical outcomes and healthcare utilization. This may limit the generalizability of our results to all people with metastatic prostate cancer.

Third, our analysis was limited to the first 5 years following randomization. Notably, approximately 60% of participants remained on enzalutamide beyond 5 years. While projections beyond progression can be made, these are less reliable than our calculations based on prospectively collected and observed healthcare costs.

Fourth, our analysis did not capture the reasons for healthcare utilization, making it difficult to determine costs with adverse effects of therapy (e.g. for falls and factures). In addition, healthcare costs incurred after disease progression; including hospitalizations (including ICU stays, HDU stays, ward stays, and emergency department presentations), subsequent lines of therapy or palliative care, were not collected, which could underestimate the long-term total healthcare costs.

Our within-trial analysis, using prospectively collected, real-world cost and health outcome data, presents a comprehensive estimate of observed real-world costs and consequences of adding enzalutamide to standard treatment in this population. The higher healthcare costs associated with enzalutamide treatment are driven by cost of the drug, longer duration on therapy, and complications including falls and fractures. The latter are at least partially preventable and if well-managed, could improve the economic and clinical benefit of longer progression-free and overall survival.

## Contributors

Chi Kin Law: Conceptualization; accessed and verified the data; formal analysis, software, methodology, visualisation, writing—original draft, and review & editing.

Martin Stockler: Conceptualization, project administration; resources, and writing—review & editing.

Andrew Martin: Data curation, accessed and verified the data (overall survival and progression free survival), writing—review & editing.

Ian Davis: Project administration, funding acquisition, investigation, resources, and writing—review & editing.

Christopher Sweeney: Project administration, funding acquisition, investigation, resources, and writing—review & editing.

Rachael Morton: Conceptualization; accessed and verified the data; supervision, investigation, methodology, writing—original draft, and review & editing.

All authors contributed to the manuscript and approved the final draft for submission.

## Data sharing statement

Requests for de-identified individual patient or aggregate data, the corresponding data dictionary with all relevant fields, and informed consent should be directed to the corresponding author and will be reviewed for data sharing by the ENZAMET trial executive committee. Decisions on whether data are to be shared will be adjudicated on the basis of the quality of questions being addressed.

## Editor note

The Lancet Group takes a neutral position with respect to territorial claims in published maps and institutional affiliations.

## Declaration of interests

Chi Kin Law served as Treasurer of the Health Services Research Association of Australia and New Zealand from 2022 to 2024 and received financial support to attend the 2024 Health Services Research Conference in Brisbane. He also served as an individual consultant for the Asian Development Bank (ADB) and received direct payment for this role. In addition, he is a shareholder of CSL Limited, a company listed on the Australian Securities Exchange (ASX).

Martin R. Stockler received research grants from Astellas, Amgen, AstraZeneca, Bionomics, Bristol Myers Squibb, Celgene, Medivation, Merck Sharp & Dohme, Pfizer, Roche, Sanofi, and Tilray. All grant funding was paid to his institution.

Andrew J. Martin is an unpaid member of the ANZUP Scientific Advisory Committee and has served on ANZUP Data and Safety Monitoring Boards (DSMBs). These roles were unrelated to the current work.

Christopher Sweeney received research grants from Bayer, Astellas, Pfizer, and Janssen, which are paid to institution. He was also a consultant and received direct payment for Bayer, Astellas, Janssen, Pfizer, Novartis, Genentech/Roche, BMS, Lilly, Novartis, Astra Zeneca, and Advancell. He has patents for Parthenolide as a cancer treatment (Indiana University); Dimethylaminoparthenolide as a cancer treatment (Leuchemix); the combination of abiraterone and cabozantinib (Dana-Farber Cancer Institute, DFCI); FRAS1 SNP and tristetraprolin as biomarkers of lethal prostate cancer (DFCI); KDM5D and NEDD as biomarkers of lethal prostate cancer (DFCI); and Parthenolide as a radioprotectant (DFCI/University of Adelaide).

Ian Davis is the unpaid Director and Chair of the Board of the ANZUP Cancer Trials Group. He has also served on advisory boards for AstraZeneca, Bayer, Roche, Telix, Astellas, and Merck Sharp & Dohme (MSD), with no personal remuneration received.

Rachael L Morton declares no competing interests.
