# Supplementary material for: Cost-effectiveness of adding enzalutamide vs standard non-steroidal antiandrogen drugs for patients with metastatic hormone-sensitive prostate cancer undergoing standard first-line therapy: a within-trial, secondary end-point analysis of the ENZAMET phase 3, international randomized trial (ANZUP 1304)
Source: eClinicalMedicine. 2026 Oct 5;100:104207. doi: 10.1016/j.eclinm.2026.104207 (PMC13673054; doi:10.1016/j.eclinm.2026.104207)
Supplement: Supplementary file 1 [file mmc1.docx]

Captions for supplementary material file

| **Content** | **Page** |
| --- | --- |
| Appendix 1. Publicly available pricing for enzalutamide | 2 |
| Appendix 2. Handling missing data from resource utilization in the trial record | 3-4 |
| Appendix 3: Data analysis using the Dose Modification from ENZAMET trial records | 5-6 |
| Appendix 4: Methods for probabilistic sensitivity analysis | 7 |
| Table S1. CRF completion rates by treatment allocation | 3 |
| Table S2.1 Comparison of the number of ICU days between treatment arms | 8 |
| Table S2.2 Comparison of HDU days between treatment arms | 9 |
| Table S2.3 Comparison of general ward admitted days between treatment arms | 10 |
| Table S2.4 Comparison of the number of ED presentations between treatment arms | 11 |
| Table S2.5 Comparison of the number of MBS claims for on-treatment participants between treatment arms | 12 |
| Table S2.6 Comparison of the number of MBS claims for off-treatment participants between treatment arms | 13 |
| Table S2.7 Comparison of the number of PBS claims for on-treatment participants between treatment arms | 14 |
| Table S2.8 Comparison of PBS claims for off-treatment participants between treatment arms | 15 |
| Table S3.1 Number of MBS claims a by Broad Type of Service (BTOS) classification and study status | 16 |
| Table S3.2 MBS costs by Broad Type of Service (BTOS) classification and study status | 16 |
| Table S4.1 Prescribed medicines claims by drug classification and study status | 17 |
| Table S4.2 Prescribed medicines costs a by drug classification and study status | 19-20 |
| Table S5.1. Mean values, standard deviations, and estimated distribution parameters used in the probabilistic sensitivity analysis (PSA) | 21 |
| Table S5.2. PSA summary statistics: Enzalutamide vs. NSAA based on 100,000 Monte Carlo simulations | 22 |
| Figure S1: ENZAMET trial sites by country | 23 |

**Appendix 1. Publicly available pricing for enzalutamide**

| Country | Cost per 40mg enzalutamide tablet (unless specified) | Date | Source | Cost per tablet (AUD) |
| --- | --- | --- | --- | --- |
| Australia | AUD $3,536.96 (112 tablets) | 2022 | Pharmaceuticals Benefits Scheme, Australia ^a^ | $32 |
| United States | USD $65.91 to $130 | 2022 | Knowledge Ecology International Online ^b^ | $99 to $196 |
| Japan | USD $21.64 ^e^ | 2021 | Okumura et al (2021); cited KEI ^c^ | $33 |
| Canada | USD $22.3 ^e^ | 2021 | Knowledge Ecology International Online ^b^ | $35 |
| Denmark | USD $44.58 ^e^ | 2021 | Knowledge Ecology International Online ^b^ | $67 |
| United Kingdom | GBP £24.42 ^f^ | 2015 | NICE ^d^ | $65 |
| Developing countries (Generic versions) | USD $ 2.31^e^ | 2021 | WHO Model Lists of Essential Medicine ^g^ | $3 |

Abbreviations: AUD, Australian dollar; GBP, Great Britain Pound; KEI, Knowledge Ecology International Online; mg, milligram; NICE, National Institute for Health and Care Excellence; USD, United States dollar; WHO, World Health Organization

Footnotes:

^a^ PBS item: 10174L; Refers to the PBS dispensed price for maximum quantity (DPMQ), which is 112 tablets

^b^ <https://www.keionline.org/xtandi2021>

^c^ Okumura H, Inoue S, Naidoo S, Holmstrom S, Akaza H. Cost-effectiveness analysis of enzalutamide for patients with chemotherapy-naïve metastatic castration-resistant prostate cancer in Japan. Japanese Journal of Clinical Oncology. 2021 Aug;51(8):1319-29.

^d^ National Institute for Health and Care Excellence. Final Appraisal Determination Enzalutamide for treating metastatic hormone-relapsed prostate cancer before chemotherapy is indicated. <https://www.nice.org.uk/guidance/ta377/documents/final-appraisal-determination-document#:~:text=2.3%20The%20cost%20(list%20price,with%20the%20Department%20of%20Health>.

^e^ 2023 average exchange rate of 0.6645 USD to 1 AUD. <https://www.exchangerates.org.uk/AUD-USD-spot-exchange-rates-history-2023.html>

^f^ 2023 average exchange rate of 0.5344 GBP to 1 AUD. <https://www.exchangerates.org.uk/AUD-GBP-spot-exchange-rates-history-2023.html>

^g^ World Health Organization Model Lists of Essential Medicine. Enzalutamide. <https://list.essentialmeds.org/recommendations/971>

**Appendix 2: Handling missing data from resource utilization in the trial record**

In this economic evaluation, trial participants' utilization of hospital services was derived from trial records collected through electronic case report forms (CRF). To determine the most appropriate approach for handling missing data, we examined the pattern of missingness by treatment allocation over the 5-year follow-up period.

Table S1 presents the CRF completion rates for intensive care unit (ICU), high dependency unit (HDU), general ward stays, and emergency department (ED) presentations. Overall, the completion rate remained above 80% throughout the follow-up period, although it showed a gradual decline over time. In addition, the completion rate was slightly higher in the enzalutamide arm than in the NSAA arm.

Table S1. CRF completion rates by treatment allocation

| Follow-up visit number | Years after randomisation | Enzalutamide complete data | | | | NSAA complete data | | | |
| --- | --- | --- | --- | --- | --- | --- | --- | --- | --- |
|  |  | ICU | HDU | GEN | ED | ICU | HDU | GEN | ED |
| 1 | 0.08 | 0.98 | 0.98 | 0.98 | 0.91 | 0.97 | 0.97 | 0.97 | 0.89 |
| 2 | 0.23 | 0.98 | 0.98 | 0.98 | 0.90 | 0.98 | 0.98 | 0.98 | 0.90 |
| 3 | 0.46 | 0.95 | 0.95 | 0.95 | 0.87 | 0.93 | 0.93 | 0.93 | 0.84 |
| 4 | 0.69 | 0.97 | 0.97 | 0.97 | 0.90 | 0.91 | 0.91 | 0.91 | 0.82 |
| 5 | 0.92 | 0.95 | 0.95 | 0.95 | 0.87 | 0.91 | 0.91 | 0.91 | 0.81 |
| 6 | 1.15 | 0.96 | 0.96 | 0.96 | 0.88 | 0.90 | 0.90 | 0.90 | 0.81 |
| 7 | 1.38 | 0.97 | 0.97 | 0.97 | 0.89 | 0.91 | 0.91 | 0.91 | 0.82 |
| 8 | 1.61 | 0.97 | 0.97 | 0.97 | 0.88 | 0.94 | 0.94 | 0.94 | 0.85 |
| 9 | 1.84 | 0.96 | 0.96 | 0.96 | 0.88 | 0.92 | 0.92 | 0.92 | 0.83 |
| 10 | 2.07 | 0.97 | 0.97 | 0.97 | 0.90 | 0.90 | 0.90 | 0.90 | 0.81 |
| 11 | 2.30 | 0.96 | 0.96 | 0.96 | 0.88 | 0.92 | 0.92 | 0.92 | 0.83 |
| 12 | 2.53 | 0.96 | 0.96 | 0.96 | 0.88 | 0.92 | 0.92 | 0.92 | 0.83 |
| 13 | 2.76 | 0.95 | 0.95 | 0.95 | 0.87 | 0.93 | 0.93 | 0.93 | 0.84 |
| 14 | 2.99 | 0.96 | 0.96 | 0.96 | 0.87 | 0.93 | 0.93 | 0.93 | 0.84 |
| 15 | 3.22 | 0.96 | 0.96 | 0.96 | 0.88 | 0.95 | 0.95 | 0.95 | 0.85 |
| 16 | 3.45 | 0.96 | 0.96 | 0.96 | 0.87 | 0.94 | 0.94 | 0.94 | 0.86 |
| 17 | 3.68 | 0.96 | 0.96 | 0.96 | 0.87 | 0.91 | 0.91 | 0.91 | 0.83 |
| 18 | 3.91 | 0.96 | 0.96 | 0.96 | 0.87 | 0.95 | 0.95 | 0.95 | 0.88 |
| 19 | 4.14 | 0.96 | 0.96 | 0.96 | 0.88 | 0.95 | 0.95 | 0.95 | 0.87 |
| 20 | 4.37 | 0.95 | 0.95 | 0.95 | 0.88 | 0.92 | 0.92 | 0.92 | 0.86 |
| 21 | 4.60 | 0.98 | 0.98 | 0.98 | 0.91 | 0.94 | 0.94 | 0.94 | 0.89 |
| 22 | 4.83 | 0.94 | 0.94 | 0.94 | 0.87 | 0.89 | 0.89 | 0.89 | 0.85 |
| 23 | 5.06 | 0.84 | 0.84 | 0.84 | 0.78 | 0.82 | 0.82 | 0.81 | 0.79 |

Abbreviations: ED, emergency department; GEN, general ward; HDU, high dependency unit; ICU, intensive care unit; NSAA, nonsteroidal anti-androgen

These findings suggest that an available-case analysis may introduce bias in the estimated mean hospital healthcare utilization and its associated standard deviation due to differential and increasing missingness over time. Missing data in the CRFs were handled using multiple Imputation (MI) under the Missing at Random (MAR) assumption. The Fully Conditional Specification (FCS) method, also known as Multiple Imputation by Chained Equations (MICE), with predictive mean matching was used to accommodate the non-normal, zero-inflated distribution typical of healthcare utilization data.

The imputation model was stratified by treatment allocation to allow arm-specific covariance structures, and included all longitudinal resource use variables alongside treatment duration as an auxiliary predictor of missingness, with 20 imputed datasets generated.

Pooled standard deviations for each visit and treatment arm were derived by applying Rubin’s rules ^1^: the total variance for each parameter was calculated as the sum of the average within-imputation variance across the 20 datasets and the between-imputation variance (the variance of the parameter estimates across the imputed datasets). The pooled standard deviation was then obtained as the square root of this total variance.

**References**

1. Burgess S, White IR, Resche‐Rigon M, Wood AM. Combining multiple imputation and meta‐analysis with individual participant data. Statistics in medicine. 2013 Nov 20;32(26):4499-514.

**Appendix 3: Data analysis using the Dose Modification from ENZAMET trial records**

1. **Dataset overview**
   - An updated dataset was received from ENZAMET on 9 April 2025, which served as the basis for the main outcome analysis (Sweeney et al., 2023).
   - The dataset includes 978 records:
     i. 5 records contain a stopping date that falls after the trial cut-off date (19 January 2022).
     ii. 164 records contain only participant IDs, with all other variables blank.
     iii. These 169 records were excluded from this analysis
   - The remaining 809 records provide dose modification information for 398 enzalutamide participants.
   - 148 records indicate a dosage reduction (variable: itmtrtmoddose), involving 96 participants (consistent with the main outcome results).
   - 140 records show a reduced restart dose (defined as less than 4 tablets per day or 160 mg), involving 79 participants.
   - 97 records (69 participants) experienced both a dosage reduction and a reduced restart dose.
   - 10 participants had a reduced restart dose without a corresponding record of prior dosage reduction:
     i. Patients: #25, #302, #436, #758, #867, #895, #1004, #1006, #1098, #1099
2. **Records with valid stopping dates**
   - 60 records did not report a treatment stopping date and were excluded from this analysis;
   - The remaining 749 records (391 participants) contained a valid stopping date for study treatment.
3. **Records with valid restart dates**

A total of 387 records (188 participants) included valid restart dates, enabling calculation of the duration of treatment disruption.

- Patient 1004 had two overlapping records:
  i. Record 1: 5 Jun 2018 to 18 Jun 2018
  ii. Record 2: 11 Jun 2018 to 17 Jun 2018
  iii. Decision: Record 2 was excluded. Patient 1004 was considered off treatment for 13 days
- For the remaining 386 records, total disruption time was 3,537 patient-days, equivalent to a reduction of 14,148 enzalutamide tablets

4. **Disruptions before end of study treatment or trial cutoff**
   - An additional 3,868 patient-days of treatment disruption were recorded among 294 participants who ceased treatment prior to end of study treatment or trial cut-off (19 January 2022)
   - This corresponds to an additional reduction of 15,472 tablets
5. **Reduced dosage and its impact on enzalutamide use**
   - Among the 386 records with valid restart dates, 139 records (involving 79 patients) reported a reduced restart dose (i.e. less than 4 tablets a day).
   - Several trial records did not record complete dosing information and we have taken the following methods and assumptions to estimate how many enzalutamide tablets were consumed:
     i. Patient 327 stopped enzalutamide between 13 Feb 2019 and 14 Feb 2019; Their restart dose in the previous and subsequent disruption was 2 tablets a day; so it was assumed they kept at this dosage level (i.e. 2 per day) when restarted on 14 Feb 2019.
     ii. Patient 436 stopped enzalutamide with missing restart dose from 27 March 2017 to 13 April 2017 and from 28 April 2017 to 5 July 2017; According to variable itmtrtmoddose, it was a “dosage reduction” in the first record, and “no change” in the second record; so it is assumed they had a dosage level of 1 tablet per day during this period of time.
     iii. Patient 574 had missing dosage from 19 June 2020 to 20 July 2020 while they had a daily dosage of 2 tablets per day before stopping study treatment. The variable “itmtrtmoddose” indicated a dose reduction when they restarted on 19 June 2020 while a dosage increase (restart dosage at 2 tablets a day) in their subsequent record on 20 July 2020. Therefore, it was expected they had a dosage level of 1 tablet per day during this period of time.
     iv. Patient 596 stopped enzalutamide on 6 June 2018. There was no restart date in this record and another record showed they stopped the drug again on 4 July 2018. It was assumed that patient 596 had no restart after 6 June 2018 until their end of study treatment on 4 July 2018.
     v. Patient 614 stopped enzalutamide on 16 June 2016. There was no restart date in this record, and another record showed they stopped the drug again on 7 November 2016. It was assumed patient 614 had no drug disruption on 16 June 2016, but consumed a daily dosage of 3 tablets until 7 November 2016.
     vi. There were two records indicating patient 617 stopped enzalutamide on 30 Nov 2016 and 7 Jan 2019 while no records indicated the patient restarted the study intervention; it was assumed patient 617 had a daily dosage of 3 tablets from 3 Nov 2016 to end of study treatment on 10 Jan 2019 (i.e. 798 days)
     vii. Patient 859 stopped enzalutamide from 24 Oct 2016 to 1 Nov 2016; it was their first disruption with a missing restart dose. In their second disruption between 9 Nov and 10 Nov 2016, their restart dose was 2 tablets per day, a dose increase according to itmtrtmoddose; it was therefore assumed their daily dose from 1 Nov 2016 to 9 Nov 2016 was 1 tablet per day.
   - The number of days from restart date to the next stop date, end of study treatment or trial cut-off (19 Jan 2022) were counted for all applicable records.
   - Dose modifications/ reductions were observed over 60,335 patient-days, representing 89,116 fewer enzalutamide tablets
6. In total, it was estimated **enzalutamide tablet consumption during the ENZAMET trial was reduced by 118,736 tablets**, due to:

- 14,148 tablets from temporary treatment disruptions
- 15,472 tablets from pre-cutoff disruptions
- 89,116 tablets from reduced dosage

This represents a **4.1% reduction** from the estimated **2.9 million tablets** that would have been used under full prescribed dosage (based on a standard dose of 4 tablets/day).

**Appendix 4. Methods for probabilistic sensitivity analysis**

In this probabilistic sensitivity analysis, the total cost for each treatment allocation was divided into nine components: (1) study drugs, (2) ICU stay, (3) HDU stay, (4) ward stay, (5) ED presentations, (6) on-treatment MBS benefits, (7) on-treatment PBS benefits, (8) off-treatment MBS benefits, and (9) off-treatment PBS benefits. For health utilities, only on-treatment EQ-5D-5L utility values were included, as participants’ off-treatment health-related quality of life (HRQoL) data were unavailable. Based on the 2012 ISPOR-SMDM report (1), Gamma distributions were assigned to cost parameters, while Beta distributions were used for EQ-5D-5L utility values. Distribution parameters were estimated from the base-case means and standard deviations using the method of moments (Table S5.1).

Gamma distribution

For a Gamma distribution parameterized by shape (α) and scale (θ), the theoretical mean is μ = α × θ and the variance is σ² (or SD²) = α × θ². Solving these equations simultaneously yields the following estimators: shape α = μ² / σ² = (μ / SD)² and scale θ = σ² / μ = SD² / μ. For Gamma variables with shape parameter α < 0.5, a Devroye transformation (2) for Gamma random variate generation was applied to improve numerical stability during simulation.

Beta distribution

For a Beta distribution parameterized by two shape parameters (α and β), the theoretical mean is μ = α / (α + β) and the variance is σ² (or SD²) = αβ / [(α + β)²(α + β + 1)]. Solving these equations simultaneously yields the following estimators: first, calculate the precision parameter κ = [μ(1 − μ) / SD²] − 1, then derive the shape parameters as α = μ × κ and β = (1 − μ) × κ.

As a result, a critical validity constraint for the Beta distribution is that the variance must satisfy σ² < μ(1 − μ); if this condition is violated, the resulting shape parameters would be non-positive and the distribution mathematically undefined.

**Table S2.1 Comparison of the number of ICU days between treatment arms**

| Trial follow-up | Months after randomization | A: Per patient days in ICU ($X_{t}$) | | B: Variance of per patient days in ICU ($S_{t}^{2}$) | | C: Average number of on-treatment participants ($P_{t}$) | | D: Average days in ICU (A x C)^*^ | | E: Variance of days in ICU (B x C^2^)^+^ | |
| --- | --- | --- | --- | --- | --- | --- | --- | --- | --- | --- | --- |
|  |  | ENZA | NSAA | ENZA | NSAA | ENZA | NSAA | ENZA | NSAA | ENZA | NSAA |
| 1 | 0.96 | 0.00 | 0.00 | 0.00 | 0.00 | 561.5 | 557.5 | 0.0 | 0.0 | 0.0 | 0.0 |
| 2 | 2.76 | 0.01 | 0.00 | 0.07 | 0.00 | 555.5 | 550 | 6.0 | 0.0 | 20161.3 | 0.0 |
| 3 | 5.52 | 0.01 | 0.01 | 0.02 | 0.02 | 541 | 529 | 4.2 | 3.1 | 5752.9 | 4928.7 |
| 4 | 8.28 | 0.00 | 0.01 | 0.00 | 0.02 | 524 | 487.5 | 1.0 | 3.3 | 531.1 | 4839.7 |
| 5 | 11.04 | 0.00 | 0.00 | 0.00 | 0.00 | 506.5 | 443.5 | 0.0 | 1.0 | 0.0 | 465.0 |
| 6 | 13.8 | 0.00 | 0.00 | 0.00 | 0.00 | 485 | 401.5 | 0.0 | 0.0 | 0.0 | 0.0 |
| 7 | 16.56 | 0.00 | 0.00 | 0.00 | 0.00 | 467 | 361 | 0.0 | 0.0 | 0.0 | 0.0 |
| 8 | 19.32 | 0.02 | 0.00 | 0.08 | 0.00 | 449.5 | 330.5 | 7.2 | 1.0 | 17039.0 | 342.4 |
| 9 | 22.08 | 0.00 | 0.00 | 0.00 | 0.00 | 431.5 | 303 | 0.0 | 0.0 | 0.0 | 0.0 |
| 10 | 24.84 | 0.02 | 0.02 | 0.12 | 0.06 | 419 | 273.5 | 10.1 | 4.2 | 21151.7 | 4603.2 |
| 11 | 27.6 | 0.01 | 0.00 | 0.02 | 0.00 | 406.5 | 249.5 | 4.1 | 1.0 | 3304.8 | 260.5 |
| 12 | 30.36 | 0.02 | 0.01 | 0.12 | 0.04 | 393 | 230 | 9.1 | 3.1 | 17922.0 | 2154.3 |
| 13 | 33.12 | 0.07 | 0.02 | 0.87 | 0.08 | 376.5 | 213.5 | 24.7 | 4.1 | 123664.8 | 3540.4 |
| 14 | 35.88 | 0.01 | 0.00 | 0.03 | 0.00 | 361 | 197.5 | 3.0 | 0.0 | 3294.6 | 0.0 |
| 15 | 38.64 | 0.00 | 0.00 | 0.00 | 0.00 | 351 | 182.5 | 0.0 | 0.0 | 0.0 | 0.0 |
| 16 | 41.4 | 0.00 | 0.00 | 0.00 | 0.00 | 340 | 173 | 0.0 | 0.0 | 0.0 | 0.0 |
| 17 | 44.16 | 0.00 | 0.00 | 0.00 | 0.00 | 329 | 165 | 0.0 | 0.0 | 0.0 | 0.0 |
| 18 | 46.92 | 0.01 | 0.01 | 0.05 | 0.03 | 319 | 154.5 | 4.1 | 2.1 | 5185.3 | 640.8 |
| 19 | 49.68 | 0.04 | 0.00 | 0.40 | 0.00 | 309 | 146.5 | 11.2 | 0.0 | 38004.0 | 0.0 |
| 20 | 52.44 | 0.03 | 0.00 | 0.12 | 0.00 | 299.5 | 141.5 | 10.2 | 0.0 | 11199.8 | 0.0 |
| 21 | 55.2 | 0.00 | 0.00 | 0.00 | 0.00 | 292 | 136.5 | 0.0 | 0.0 | 0.0 | 0.0 |
| 22 | 57.96 | 0.00 | 0.00 | 0.00 | 0.00 | 284 | 130.5 | 0.0 | 0.0 | 0.0 | 0.0 |
| 23 | 60.72 | 0.00 | 0.00 | 0.00 | 0.00 | 257.5 | 118 | 0.0 | 0.0 | 0.0 | 0.0 |

Abbreviations: ENZA, enzalutamide; ICU, intensive care unit; NSAA, nonsteroidal anti-androgen

^*^Per patient-year ICU days in first 60 months was calculated by summing the average days in ICU from trial follow-up 1 to 23 (column D – Enzalutamide=95.02; NSAA=23.09) divided by number of patient-years exposed (Enzalutamide=2001; NSAA=:1362)

^+^ Variance of per patient-year ICU days in the first 60 months was calculated by summing the variance of ICU days from trial follow-up 1 to 23 (column E – Enzalutamide= 267,211; NSAA=21,775) divided by the square form of patient-years exposed (Enzalutamide=2001^2^ ; NSAA= 1362 ^2^)

ENZA: 0.047 (Variance 0.0667) vs. NSAA: 0.017 (Variance 0.0117), t=2.584, p=0.01, df=1123. ICU = intensive care unit

**Table S2.2 Comparison of HDU days between treatment arms**

| Trial follow-up | Months after randomization | A: Per patient days in HDU ($X_{t}$) | | B: Variance of per patient days in HDU ($S_{t}^{2}$) | | C: Average number of on-treatment participants ($P_{t}$)) | | D: Average days in HDU (A x C) ^*^ | | E: Variance of days in HDU (B x C^2^) ^+^ | |
| --- | --- | --- | --- | --- | --- | --- | --- | --- | --- | --- | --- |
|  |  | ENZA | NSAA | ENZA | NSAA | ENZA | NSAA | ENZA | NSAA | ENZA | NSAA |
| 1 | 0.96 | 0.00 | 0.00 | 0.00 | 0.00 | 561.5 | 557.5 | 1.0 | 0.0 | 563.0 | 0.0 |
| 2 | 2.76 | 0.01 | 0.00 | 0.05 | 0.00 | 555.5 | 550 | 5.0 | 0.0 | 14000.9 | 0.0 |
| 3 | 5.52 | 0.01 | 0.00 | 0.02 | 0.00 | 541 | 529 | 3.1 | 0.0 | 4960.7 | 0.0 |
| 4 | 8.28 | 0.00 | 0.00 | 0.00 | 0.00 | 524 | 487.5 | 0.0 | 1.1 | 0.0 | 512.2 |
| 5 | 11.04 | 0.00 | 0.00 | 0.00 | 0.00 | 506.5 | 443.5 | 0.0 | 0.0 | 0.0 | 0.0 |
| 6 | 13.8 | 0.00 | 0.01 | 0.00 | 0.01 | 485 | 401.5 | 0.0 | 2.3 | 0.0 | 1866.1 |
| 7 | 16.56 | 0.00 | 0.00 | 0.01 | 0.00 | 467 | 361 | 2.0 | 0.0 | 1896.4 | 0.0 |
| 8 | 19.32 | 0.00 | 0.00 | 0.00 | 0.00 | 449.5 | 330.5 | 0.0 | 0.0 | 0.0 | 0.0 |
| 9 | 22.08 | 0.00 | 0.00 | 0.00 | 0.00 | 431.5 | 303 | 0.0 | 1.1 | 0.0 | 319.9 |
| 10 | 24.84 | 0.03 | 0.01 | 0.36 | 0.02 | 419 | 273.5 | 14.5 | 2.1 | 62958.0 | 1150.8 |
| 11 | 27.6 | 0.00 | 0.00 | 0.00 | 0.00 | 406.5 | 249.5 | 0.0 | 0.0 | 0.0 | 0.0 |
| 12 | 30.36 | 0.00 | 0.00 | 0.00 | 0.00 | 393 | 230 | 0.0 | 0.0 | 0.0 | 0.0 |
| 13 | 33.12 | 0.00 | 0.00 | 0.00 | 0.00 | 376.5 | 213.5 | 0.0 | 0.0 | 0.0 | 0.0 |
| 14 | 35.88 | 0.00 | 0.00 | 0.00 | 0.00 | 361 | 197.5 | 0.0 | 0.0 | 0.0 | 0.0 |
| 15 | 38.64 | 0.00 | 0.00 | 0.00 | 0.00 | 351 | 182.5 | 0.0 | 0.0 | 0.0 | 0.0 |
| 16 | 41.4 | 0.00 | 0.00 | 0.00 | 0.00 | 340 | 173 | 0.0 | 0.0 | 0.0 | 0.0 |
| 17 | 44.16 | 0.00 | 0.03 | 0.00 | 0.10 | 329 | 165 | 0.0 | 4.1 | 0.0 | 2722.5 |
| 18 | 46.92 | 0.00 | 0.00 | 0.00 | 0.00 | 319 | 154.5 | 0.0 | 0.0 | 0.0 | 0.0 |
| 19 | 49.68 | 0.02 | 0.00 | 0.12 | 0.00 | 309 | 146.5 | 6.1 | 0.0 | 11307.0 | 0.0 |
| 20 | 52.44 | 0.00 | 0.00 | 0.00 | 0.00 | 299.5 | 141.5 | 0.0 | 0.0 | 0.0 | 0.0 |
| 21 | 55.2 | 0.00 | 0.00 | 0.00 | 0.00 | 292 | 136.5 | 0.0 | 0.0 | 0.0 | 0.0 |
| 22 | 57.96 | 0.00 | 0.00 | 0.00 | 0.00 | 284 | 130.5 | 0.0 | 0.0 | 0.0 | 0.0 |
| 23 | 60.72 | 0.00 | 0.00 | 0.00 | 0.00 | 257.5 | 118 | 0.0 | 0.0 | 0.0 | 0.0 |

Abbreviations: ENZA, enzalutamide; HDU, high dependency unit; NSAA, nonsteroidal anti-androgen

^*^ Per patient-year HDU days in first 60 months was calculated by summing the average days in HDU from trial follow-up 1 to 23 (column D – Enzalutamide=31.70; NSAA=10.66) divided by number of patient-years exposed (Enzalutamide=2001; NSAA=:1362)

^+^ Variance of per patient-year HDU days in the first 60 months was calculated by summing the variance of HDU days from trial follow-up 1 to 23 (column E – Enzalutamide= 95,686; NSAA= 6572) divided by the square form of patient-years exposed (Enzalutamide=2001^2^ ; NSAA= 1362 ^2^)

ENZA: 0.016 (Variance 0.0239) vs. NSAA: 0.008 (Variance 0.0035), t=1.147, p=0.252, df=1123. HDU = high dependency unit

**Table S2.3 Comparison of general ward admitted days between treatment arms**

| Trial follow-up | Months after randomization | A: Per patient days in general ward ($X_{t}$) | | B: Variance of per patient days in general ward ($S_{t}^{2}$) | | C: Average number of on-treatment participants ($P_{t}$) | | D: Average days in general ward (A x C) ^*^ | | E: Variance of days in general ward (B x C^2^) ^+^ | |
| --- | --- | --- | --- | --- | --- | --- | --- | --- | --- | --- | --- |
|  |  | ENZA | NSAA | ENZA | NSAA | ENZA | NSAA | ENZA | NSAA | ENZA | NSAA |
| 1 | 0.96 | 0.195 | 0.191 | 1.203 | 0.048 | 561.5 | 557.5 | 109.6 | 106.4 | 379391.9 | 14781.3 |
| 2 | 2.76 | 0.250 | 0.312 | 1.257 | 0.089 | 555.5 | 550 | 138.9 | 171.8 | 387808.3 | 26911.7 |
| 3 | 5.52 | 0.385 | 0.307 | 5.976 | 0.086 | 541 | 529 | 208.4 | 162.3 | 1749051.7 | 24044.5 |
| 4 | 8.28 | 0.300 | 0.170 | 14.499 | 0.069 | 524 | 487.5 | 157.0 | 82.8 | 3981187.2 | 16416.8 |
| 5 | 11.04 | 0.276 | 0.148 | 4.805 | 0.092 | 506.5 | 443.5 | 139.6 | 65.5 | 1232567.7 | 18098.0 |
| 6 | 13.8 | 0.261 | 0.225 | 3.208 | 0.039 | 485 | 401.5 | 126.7 | 90.4 | 754547.8 | 6323.8 |
| 7 | 16.56 | 0.117 | 0.114 | 0.497 | 0.035 | 467 | 361 | 54.6 | 41.1 | 108364.9 | 4567.2 |
| 8 | 19.32 | 0.377 | 0.106 | 6.928 | 0.077 | 449.5 | 330.5 | 169.5 | 35.2 | 1399737.7 | 8421.6 |
| 9 | 22.08 | 0.098 | 0.125 | 0.648 | 0.071 | 431.5 | 303 | 42.2 | 37.8 | 120579.8 | 6479.4 |
| 10 | 24.84 | 0.251 | 0.131 | 10.368 | 0.052 | 419 | 273.5 | 105.3 | 35.8 | 1820169.9 | 3857.2 |
| 11 | 27.6 | 0.143 | 0.147 | 2.186 | 0.017 | 406.5 | 249.5 | 58.3 | 36.6 | 361222.8 | 1054.0 |
| 12 | 30.36 | 0.154 | 0.224 | 1.179 | 0.019 | 393 | 230 | 60.5 | 51.6 | 182026.4 | 1013.8 |
| 13 | 33.12 | 0.260 | 0.194 | 8.697 | 0.030 | 376.5 | 213.5 | 97.7 | 41.5 | 1232794.7 | 1378.9 |
| 14 | 35.88 | 0.355 | 0.202 | 8.747 | 0.070 | 361 | 197.5 | 128.1 | 39.8 | 1139887.7 | 2742.3 |
| 15 | 38.64 | 0.493 | 0.304 | 24.274 | 0.042 | 351 | 182.5 | 173.1 | 55.5 | 2990600.5 | 1384.0 |
| 16 | 41.4 | 0.174 | 0.216 | 2.276 | 0.045 | 340 | 173 | 59.2 | 37.4 | 263146.6 | 1354.6 |
| 17 | 44.16 | 0.131 | 0.082 | 1.292 | 0.061 | 329 | 165 | 43.3 | 13.5 | 139841.8 | 1673.7 |
| 18 | 46.92 | 0.179 | 0.175 | 2.685 | 0.013 | 319 | 154.5 | 57.0 | 27.0 | 273254.6 | 318.2 |
| 19 | 49.68 | 0.250 | 0.221 | 2.888 | 0.041 | 309 | 146.5 | 77.1 | 32.4 | 275705.5 | 883.6 |
| 20 | 52.44 | 0.280 | 0.193 | 4.151 | 0.025 | 299.5 | 141.5 | 83.8 | 27.3 | 372388.8 | 495.0 |
| 21 | 55.2 | 0.291 | 0.128 | 3.902 | 0.031 | 292 | 136.5 | 85.1 | 17.4 | 332727.5 | 583.8 |
| 22 | 57.96 | 0.215 | 0.304 | 5.120 | 0.008 | 284 | 130.5 | 61.2 | 39.7 | 412989.0 | 134.1 |
| 23 | 60.72 | 0.031 | 0.032 | 0.081 | 0.020 | 257.5 | 118 | 7.9 | 3.7 | 5368.6 | 271.7 |

Abbreviations: ENZA, enzalutamide; NSAA, nonsteroidal anti-androgen

^*^Per patient-year general ward admitted days in first 60 months was calculated by summing the average days in general ward from trial follow-up 1 to 23 (column D – Enzalutamide=2244; NSAA=1252) divided by number of patient-years exposed (Enzalutamide=2001 patient-years; NSAA=1362)

^+^ Variance of per patient-year general ward admitted days in the first 60 months was calculated by summing the variance of days in general ward from trial follow-up 1 to 23 (column E – Enzalutamide= 19,915,361; NSAA= 143,189) divided by the square form of patient-years exposed (Enzalutamide=2001^2^ ; NSAA= 1362 ^2^)

ENZA: 1.121 (Variance 4.974) vs. NSAA: 0.920 (Variance 0.077), t=2.129, p=0.033, df=1123

**Table S2.4 Comparison of the number of ED presentations between treatment arms**

| Trial follow-up | Months after randomization | A: Per patient ED (X_t_) | | B: Variance of per patient ED (S_t_) | | C: Average number of on-treatment participants (P_t_) | | D: Average ED presentations^*^ | | E: Variance of ED presentations (B x C^2^) ^+^ | |
| --- | --- | --- | --- | --- | --- | --- | --- | --- | --- | --- | --- |
|  |  | ENZA | NSAA | ENZA | NSAA | ENZA | NSAA | ENZA | NSAA | ENZA | NSAA |
| 1 | 0.96 | 0.050 | 0.041 | 0.067 | 0.048 | 561.5 | 557.5 | 28.2 | 23.1 | 21201.3 | 14781.3 |
| 2 | 2.76 | 0.050 | 0.066 | 0.059 | 0.089 | 555.5 | 550.0 | 27.7 | 36.2 | 18249.8 | 26911.7 |
| 3 | 5.52 | 0.058 | 0.057 | 0.084 | 0.086 | 541.0 | 529.0 | 31.6 | 30.3 | 24703.9 | 24044.5 |
| 4 | 8.28 | 0.035 | 0.052 | 0.039 | 0.069 | 524.0 | 487.5 | 18.2 | 25.5 | 10816.5 | 16416.8 |
| 5 | 11.04 | 0.037 | 0.070 | 0.054 | 0.092 | 506.5 | 443.5 | 18.6 | 31.0 | 13906.4 | 18098.0 |
| 6 | 13.8 | 0.052 | 0.041 | 0.074 | 0.039 | 485.0 | 401.5 | 25.4 | 16.4 | 17357.3 | 6323.8 |
| 7 | 16.56 | 0.037 | 0.036 | 0.035 | 0.035 | 467.0 | 361.0 | 17.1 | 13.1 | 7690.9 | 4567.2 |
| 8 | 19.32 | 0.035 | 0.066 | 0.043 | 0.077 | 449.5 | 330.5 | 15.7 | 21.8 | 8749.4 | 8421.6 |
| 9 | 22.08 | 0.056 | 0.046 | 0.074 | 0.071 | 431.5 | 303.0 | 24.1 | 14.0 | 13703.0 | 6479.4 |
| 10 | 24.84 | 0.049 | 0.046 | 0.067 | 0.052 | 419.0 | 273.5 | 20.6 | 12.5 | 11687.8 | 3857.2 |
| 11 | 27.6 | 0.042 | 0.017 | 0.063 | 0.017 | 406.5 | 249.5 | 17.1 | 4.3 | 10459.6 | 1054.0 |
| 12 | 30.36 | 0.052 | 0.019 | 0.055 | 0.019 | 393.0 | 230.0 | 20.4 | 4.5 | 8545.3 | 1013.8 |
| 13 | 33.12 | 0.047 | 0.031 | 0.062 | 0.030 | 376.5 | 213.5 | 17.8 | 6.6 | 8731.4 | 1378.9 |
| 14 | 35.88 | 0.054 | 0.048 | 0.145 | 0.070 | 361.0 | 197.5 | 19.6 | 9.5 | 18831.8 | 2742.3 |
| 15 | 38.64 | 0.051 | 0.043 | 0.067 | 0.042 | 351.0 | 182.5 | 17.9 | 7.9 | 8231.2 | 1384.0 |
| 16 | 41.4 | 0.058 | 0.034 | 0.087 | 0.045 | 340.0 | 173.0 | 19.7 | 6.0 | 10077.4 | 1354.6 |
| 17 | 44.16 | 0.059 | 0.038 | 0.126 | 0.061 | 329.0 | 165.0 | 19.5 | 6.2 | 13674.9 | 1673.7 |
| 18 | 46.92 | 0.049 | 0.013 | 0.406 | 0.013 | 319.0 | 154.5 | 15.7 | 2.1 | 41268.5 | 318.2 |
| 19 | 49.68 | 0.022 | 0.043 | 0.022 | 0.041 | 309.0 | 146.5 | 6.9 | 6.3 | 2094.9 | 883.6 |
| 20 | 52.44 | 0.020 | 0.025 | 0.027 | 0.025 | 299.5 | 141.5 | 5.9 | 3.6 | 2406.0 | 495.0 |
| 21 | 55.2 | 0.060 | 0.016 | 0.064 | 0.031 | 292.0 | 136.5 | 17.4 | 2.1 | 5483.1 | 583.8 |
| 22 | 57.96 | 0.029 | 0.008 | 0.037 | 0.008 | 284.0 | 130.5 | 8.3 | 1.0 | 2947.5 | 134.1 |
| 23 | 60.72 | 0.026 | 0.020 | 0.025 | 0.020 | 257.5 | 118.0 | 6.6 | 2.3 | 1663.3 | 271.7 |

Abbreviations: ED, emergency department; ENZA, enzalutamide; NSAA, nonsteroidal anti-androgen

^*^Per patient-year ED presentations in first 60 months was calculated by summing the average ED presentations from trial follow-up 1 to 23 (column D – Enzalutamide=420.14; NSAA=286.22) divided by number of patient-years exposed (Enzalutamide=2001; NSAA=1362)

^+^ Variance of per patient-year ED presentations in the first 60 months was calculated by summing the variance of ED presentations from trial follow-up 1 to 23 (column E – Enzalutamide= 282,481; NSAA=143,189) divided by the square form of patient-years exposed (Enzalutamide=2001^2^ ; NSAA= 1362 ^2^)

ENZA: 0.210 (Variance 0.071) vs. NSAA: 0.210 (Variance 0.772), t=0.011, p=0.991, df=1123. ED = Emergency Department

**Table S2.5 Comparison of the number of MBS claims for on-treatment participants between treatment arms**

| Trial follow-up | Months after randomization | A: Per patient claims (X_t_)* | | B: Variance of per patient claims (S_t_)* | | C: Average number of on-treatment participants (P_t_) | | D: Average claims (A x C) ^+^ | |
| --- | --- | --- | --- | --- | --- | --- | --- | --- | --- |
|  |  | ENZA | NSAA | ENZA | NSAA | ENZA | NSAA | ENZA | NSAA |
| 1 | 0.96 | 22.8 | 22.3 | 170.8 | 149.8 | 561.5 | 557.5 | 12822 | 12449 |
| 2 | 2.76 | 16.6 | 16.7 | 146.6 | 119.0 | 555.5 | 550.0 | 9247 | 9198 |
| 3 | 5.52 | 12.7 | 12.1 | 76.2 | 63.3 | 541.0 | 529.0 | 6893 | 6401 |
| 4 | 8.28 | 10.7 | 11.9 | 51.0 | 50.5 | 524.0 | 487.5 | 5608 | 5787 |
| 5 | 11.04 | 11.6 | 13.0 | 54.2 | 137.3 | 506.5 | 443.5 | 5862 | 5770 |
| 6 | 13.8 | 11.5 | 12.2 | 61.8 | 94.9 | 485.0 | 401.5 | 5577 | 4902 |
| 7 | 16.56 | 11.8 | 12.9 | 116.9 | 135.2 | 467.0 | 361.0 | 5525 | 4664 |
| 8 | 19.32 | 12.1 | 13.0 | 69.7 | 158.5 | 449.5 | 330.5 | 5434 | 4295 |
| 9 | 22.08 | 12.4 | 12.4 | 97.7 | 80.4 | 431.5 | 303.0 | 5360 | 3749 |
| 10 | 24.84 | 12.6 | 12.5 | 246.2 | 101.3 | 419.0 | 273.5 | 5299 | 3412 |
| 11 | 27.6 | 13.7 | 11.9 | 321.6 | 69.1 | 406.5 | 249.5 | 5576 | 2964 |
| 12 | 30.36 | 11.6 | 11.9 | 60.0 | 81.4 | 393.0 | 230.0 | 4562 | 2731 |
| 13 | 33.12 | 11.9 | 11.8 | 93.9 | 67.5 | 376.5 | 213.5 | 4495 | 2526 |
| 14 | 35.88 | 11.0 | 12.5 | 49.5 | 190.2 | 361.0 | 197.5 | 3969 | 2462 |
| 15 | 38.64 | 11.3 | 13.1 | 44.4 | 79.1 | 351.0 | 182.5 | 3971 | 2385 |
| 16 | 41.4 | 11.9 | 12.3 | 53.9 | 106.4 | 340.0 | 173.0 | 4055 | 2130 |
| 17 | 44.16 | 13.6 | 13.4 | 272.9 | 305.6 | 329.0 | 165.0 | 4465 | 2215 |
| 18 | 46.92 | 12.8 | 12.0 | 119.8 | 63.9 | 319.0 | 154.5 | 4099 | 1848 |
| 19 | 49.68 | 13.1 | 13.3 | 219.4 | 232.6 | 309.0 | 146.5 | 4033 | 1951 |
| 20 | 52.44 | 12.6 | 11.6 | 114.8 | 68.7 | 299.5 | 141.5 | 3770 | 1644 |
| 21 | 55.2 | 12.1 | 12.8 | 88.6 | 142.5 | 292.0 | 136.5 | 3525 | 1745 |
| 22 | 57.96 | 11.4 | 11.5 | 47.3 | 71.0 | 284.0 | 130.5 | 3242 | 1502 |
| 23 | 60.72 | 11.0 | 12.2 | 40.2 | 94.7 | 257.5 | 118.0 | 2838 | 1442 |

Abbreviations: ENZA, enzalutamide; MBS, Medicare Benefits Schedule; NSAA, nonsteroidal anti-androgen

*Based on 504 consenting Australian participants with linked MBS claims

^+^Per patient-year MBS claims in first 60 months was calculated by summing the average MBS claims from trial follow-up 1 to 23 (column D – Enzalutamide= 120,227; NSAA= 88,172) divided by number of patient-years exposed (Enzalutamide=2001; NSAA=1362)

ENZA: 60 vs. NSAA: 65. MBS = Medicare Benefits Schedule (e.g. diagnostic tests, clinician visits, procedures)

**Table S2.6 Comparison of the number of MBS claims for off-treatment participants between treatment arms**

| Trial follow-up | Months after randomization | A: Per patient claims (X_t_)* | | B: Variance of per patient claims (S_t_)* | | C: Average number of off-treatment participants (P_t_) | | D: Average claims (A x C) ^+^ | |
| --- | --- | --- | --- | --- | --- | --- | --- | --- | --- |
|  |  | ENZA | NSAA | ENZA | NSAA | ENZA | NSAA | ENZA | NSAA |
| 1 | 0.96 | 3.0 | 34.0 | - | - | 1.5 | 2 | 5 | 68 |
| 2 | 2.76 | 17.0 | 18.2 | 180.0 | 148.4 | 6.5 | 6 | 111 | 109 |
| 3 | 5.52 | 19.0 | 20.8 | 266.0 | 278.7 | 19 | 25 | 361 | 521 |
| 4 | 8.28 | 21.2 | 23.9 | 234.1 | 488.6 | 32 | 62.5 | 680 | 1492 |
| 5 | 11.04 | 29.2 | 24.9 | 763.7 | 406.1 | 41 | 98 | 1198 | 2439 |
| 6 | 13.8 | 34.0 | 22.1 | 732.2 | 343.1 | 53.5 | 126.5 | 1819 | 2791 |
| 7 | 16.56 | 26.7 | 23.8 | 274.8 | 797.8 | 66 | 152.5 | 1762 | 3630 |
| 8 | 19.32 | 27.1 | 19.1 | 529.0 | 180.4 | 77.5 | 171 | 2098 | 3271 |
| 9 | 22.08 | 29.9 | 18.8 | 863.3 | 141.0 | 84.5 | 182.5 | 2530 | 3428 |
| 10 | 24.84 | 28.0 | 21.2 | 622.2 | 286.3 | 83.5 | 196 | 2338 | 4150 |
| 11 | 27.6 | 30.3 | 24.0 | 1155.0 | 1088.1 | 85 | 206.5 | 2575 | 4951 |
| 12 | 30.36 | 31.8 | 18.9 | 1305.1 | 162.7 | 89.5 | 212 | 2842 | 4011 |
| 13 | 33.12 | 26.2 | 22.9 | 519.5 | 863.5 | 96 | 216.5 | 2516 | 4963 |
| 14 | 35.88 | 19.0 | 24.2 | 164.5 | 801.5 | 99 | 218.5 | 1885 | 5298 |
| 15 | 38.64 | 18.3 | 23.5 | 231.1 | 676.9 | 97.5 | 218.5 | 1781 | 5129 |
| 16 | 41.4 | 20.3 | 22.7 | 246.8 | 1413.3 | 99 | 214.5 | 2014 | 4870 |
| 17 | 44.16 | 20.6 | 16.9 | 416.9 | 145.8 | 100.5 | 207 | 2069 | 3495 |
| 18 | 46.92 | 18.3 | 20.0 | 14.4 | 23.4 | 101 | 202 | 1848 | 4042 |
| 19 | 49.68 | 21.4 | 23.2 | 22.4 | 20.8 | 101 | 200.5 | 2157 | 4650 |
| 20 | 52.44 | 21.3 | 24.8 | 23.1 | 25.8 | 100.5 | 198.5 | 2145 | 4932 |
| 21 | 55.2 | 24.1 | 19.8 | 20.8 | 14.7 | 96 | 197 | 2316 | 3903 |
| 22 | 57.96 | 20.9 | 15.3 | 16.8 | 11.8 | 91.5 | 195 | 1908 | 2980 |
| 23 | 60.72 | 19.2 | 15.9 | 17.4 | 13.2 | 90 | 176 | 1732 | 2804 |

Abbreviations: ENZA, enzalutamide; MBS, Medicare Benefits Schedule; NSAA, nonsteroidal anti-androgen

*Based on 504 consenting Australian participants with linked MBS claims

^+^Per patient-year MBS claims in first 60 months was calculated by summing the average MBS claims from trial follow-up 1 to 23 (column D – Enzalutamide= 40,690; NSAA= 77,927) divided by number of patient-years exposed (Enzalutamide=393; NSAA=847):

ENZA: 104 vs. NSAA: 92.. MBS = Medicare Benefits Schedule (e.g. diagnostic tests, clinician visits, procedures)

**Table S2.7 Comparison of the number of PBS claims for on-treatment participants between treatment arms**

| Trial follow-up | Months after randomization | A: Per patient claims (X_t_)* | | B: Variance of per patient claims (S_t_)* | | C: Average number of on-treatment participants (P_t_) | | D: Average claims (A x C) ^+^ | |
| --- | --- | --- | --- | --- | --- | --- | --- | --- | --- |
|  |  | ENZA | NSAA | ENZA | NSAA | ENZA | NSAA | ENZA | NSAA |
| 1 | 0.96 | 11.3 | 13.8 | 74.3 | 65.0 | 561.5 | 557.5 | 6328 | 7699 |
| 2 | 2.76 | 9.7 | 11.9 | 68.0 | 54.8 | 555.5 | 550.0 | 5399 | 6545 |
| 3 | 5.52 | 9.2 | 10.3 | 59.1 | 48.2 | 541.0 | 529.0 | 4961 | 5470 |
| 4 | 8.28 | 8.9 | 11.2 | 64.5 | 57.7 | 524.0 | 487.5 | 4679 | 5436 |
| 5 | 11.04 | 8.8 | 10.5 | 52.8 | 37.9 | 506.5 | 443.5 | 4457 | 4657 |
| 6 | 13.8 | 9.4 | 10.4 | 63.2 | 53.1 | 485.0 | 401.5 | 4549 | 4180 |
| 7 | 16.56 | 9.1 | 10.6 | 64.5 | 46.6 | 467.0 | 361.0 | 4245 | 3819 |
| 8 | 19.32 | 9.1 | 10.6 | 58.5 | 44.0 | 449.5 | 330.5 | 4068 | 3517 |
| 9 | 22.08 | 9.6 | 10.7 | 68.0 | 51.0 | 431.5 | 303.0 | 4151 | 3251 |
| 10 | 24.84 | 9.3 | 10.8 | 58.7 | 50.1 | 419.0 | 273.5 | 3876 | 2940 |
| 11 | 27.6 | 9.4 | 10.5 | 61.6 | 57.6 | 406.5 | 249.5 | 3829 | 2627 |
| 12 | 30.36 | 9.3 | 10.7 | 63.8 | 46.6 | 393.0 | 230.0 | 3663 | 2461 |
| 13 | 33.12 | 9.9 | 10.1 | 65.0 | 42.6 | 376.5 | 213.5 | 3712 | 2165 |
| 14 | 35.88 | 9.8 | 11.1 | 63.5 | 64.3 | 361.0 | 197.5 | 3541 | 2184 |
| 15 | 38.64 | 10.0 | 11.2 | 73.8 | 55.7 | 351.0 | 182.5 | 3496 | 2035 |
| 16 | 41.4 | 10.1 | 9.8 | 70.9 | 52.5 | 340.0 | 173.0 | 3424 | 1697 |
| 17 | 44.16 | 10.1 | 10.5 | 66.1 | 60.6 | 329.0 | 165.0 | 3333 | 1739 |
| 18 | 46.92 | 9.9 | 10.0 | 72.3 | 41.5 | 319.0 | 154.5 | 3158 | 1543 |
| 19 | 49.68 | 10.1 | 10.2 | 69.0 | 53.9 | 309.0 | 146.5 | 3109 | 1500 |
| 20 | 52.44 | 10.5 | 9.4 | 62.9 | 37.0 | 299.5 | 141.5 | 3139 | 1329 |
| 21 | 55.2 | 10.2 | 9.8 | 76.4 | 35.4 | 292.0 | 136.5 | 2967 | 1332 |
| 22 | 57.96 | 10.7 | 9.4 | 79.7 | 29.2 | 284.0 | 130.5 | 3030 | 1229 |
| 23 | 60.72 | 10.7 | 9.8 | 92.5 | 40.4 | 257.5 | 118.0 | 2753 | 1151 |

Abbreviations: ENZA, enzalutamide; PBS, Pharmaceuticals Benefits Scheme; NSAA, nonsteroidal anti-androgen

*Based on 500 consenting Australian participants with linkage to PBS claims

^+^Per patient-year PBS claims in first 60 months was calculated by summing the average PBS claims from trial follow-up 1 to 23 (column D – Enzalutamide= 89,867; NSAA= 70,506) divided by number of patient-years exposed (Enzalutamide=2001; NSAA=1362)

ENZA: 45 vs. NSAA: 52. PBS = Pharmaceutical Benefits Scheme (prescribed medicines)

**Table S2.8 Comparison of PBS claims for off-treatment participants between treatment arms**

| Trial follow-up | Months after randomization | A: Per patient claims (X_t_) | | B: Variance of per patient claims (S_t_) | | C: Average number of off-treatment participants (P_t_) | | D: Average claims (A x C) ^+^ | |
| --- | --- | --- | --- | --- | --- | --- | --- | --- | --- |
|  |  | ENZA | NSAA | ENZA | NSAA | ENZA | NSAA | ENZA | NSAA |
| 1 | 0.96 | 3.0 | 3.5 | - | 4.5 | 1.5 | 2 | 5 | 7 |
| 2 | 2.76 | 12.3 | 8.1 | 53.9 | 28.1 | 6.5 | 6 | 80 | 49 |
| 3 | 5.52 | 10.6 | 12.4 | 127.7 | 73.5 | 19 | 25 | 200 | 309 |
| 4 | 8.28 | 12.3 | 15.3 | 48.9 | 106.3 | 32 | 62.5 | 394 | 954 |
| 5 | 11.04 | 18.7 | 15.8 | 307.8 | 117.1 | 41 | 98 | 768 | 1544 |
| 6 | 13.8 | 18.5 | 11.9 | 204.3 | 78.5 | 53.5 | 126.5 | 989 | 1505 |
| 7 | 16.56 | 16.5 | 13.9 | 92.4 | 87.7 | 66 | 152.5 | 1088 | 2115 |
| 8 | 19.32 | 16.5 | 14.1 | 156.8 | 98.5 | 77.5 | 171 | 1276 | 2416 |
| 9 | 22.08 | 16.2 | 13.2 | 127.4 | 61.5 | 84.5 | 182.5 | 1371 | 2414 |
| 10 | 24.84 | 17.0 | 14.1 | 170.3 | 80.7 | 83.5 | 196 | 1417 | 2769 |
| 11 | 27.6 | 15.5 | 14.9 | 166.3 | 110.7 | 85 | 206.5 | 1320 | 3085 |
| 12 | 30.36 | 17.5 | 15.1 | 211.7 | 118.3 | 89.5 | 212 | 1564 | 3210 |
| 13 | 33.12 | 14.1 | 15.6 | 74.8 | 119.8 | 96 | 216.5 | 1356 | 3367 |
| 14 | 35.88 | 12.3 | 15.1 | 64.4 | 119.5 | 99 | 218.5 | 1219 | 3299 |
| 15 | 38.64 | 12.0 | 14.5 | 78.2 | 84.8 | 97.5 | 218.5 | 1172 | 3166 |
| 16 | 41.4 | 11.8 | 15.1 | 68.9 | 96.8 | 99 | 214.5 | 1164 | 3233 |
| 17 | 44.16 | 11.8 | 14.8 | 72.2 | 123.7 | 100.5 | 207 | 1190 | 3053 |
| 18 | 46.92 | 12.1 | 13.9 | 78.5 | 94.9 | 101 | 202 | 1220 | 2798 |
| 19 | 49.68 | 12.6 | 13.0 | 96.2 | 83.5 | 101 | 200.5 | 1268 | 2602 |
| 20 | 52.44 | 12.7 | 14.9 | 99.0 | 116.8 | 100.5 | 198.5 | 1275 | 2966 |
| 21 | 55.2 | 12.9 | 13.9 | 94.2 | 103.2 | 96 | 197 | 1236 | 2728 |
| 22 | 57.96 | 14.3 | 13.5 | 126.4 | 103.1 | 91.5 | 195 | 1311 | 2640 |
| 23 | 60.72 | 13.0 | 13.1 | 75.1 | 122.2 | 90 | 176 | 1170 | 2307 |

Abbreviations: ENZA, enzalutamide; PBS, Pharmaceuticals Benefits Scheme; NSAA, nonsteroidal anti-androgen

*Based on 500 consenting Australian participants with linked PBS claims

Per patient-year PBS claims in first 60 months was calculated by summing the average PBS claims from trial follow-up 1 to 23 (column D – Enzalutamide= 24,053; NSAA= 52,536) divided by number of patient-years exposed (Enzalutamide=393; NSAA=847):

ENZA: 61 vs. NSAA: 62. PBS = Pharmaceutical Benefits Scheme (prescribed medicines)

**Table S3.1 Number of MBS claims ^a^ by Broad Type of Service (BTOS) classification and study status**

| Broad Type of Service (BTOS) | On study treatment | | | | | | Off study treatment | | | | | | |
| --- | --- | --- | --- | --- | --- | --- | --- | --- | --- | --- | --- | --- | --- |
|  | ENZA | | NSAA | | All | | | ENZA | | NSAA | | All | |
|  | Claims | % | Claims | % | Claims | % | | Claims | % | Claims | % | Claims | % |
|  |  |  |  |  |  |  | |  |  |  |  |  |  |
| Diagnostic Imaging Services | 2985 | 4.5 | 2397 | 5.7 | 5382 | 5.0 | | 1430 | 6.2 | 2217 | 6.0 | 3647 | 6.1 |
| Diagnostic Procedures and Investigations | 1072 | 1.6 | 687 | 1.6 | 1759 | 1.6 | | 247 | 1.1 | 351 | 0.9 | 598 | 1.0 |
| Pathology Services | 33203 | 50.0 | 21517 | 51.5 | 54720 | 50.5 | | 10897 | 47.5 | 18798 | 50.8 | 29695 | 49.5 |
| Professional Attendances | 22363 | 33.7 | 13278 | 31.8 | 35641 | 32.9 | | 7234 | 31.5 | 11220 | 30.3 | 18454 | 30.8 |
| Therapeutic Procedures | 4795 | 7.2 | 3033 | 7.3 | 7828 | 7.2 | | 2742 | 12.0 | 3754 | 10.1 | 6496 | 10.8 |
| Miscellaneous Services ^b^ | 2039 | 3.1 | 881 | 2.1 | 2920 | 2.7 | | 395 | 1.7 | 658 | 1.8 | 1053 | 1.8 |
|  |  |  |  |  |  |  | |  |  |  |  |  |  |
| Total | 66457 | 100 | 41793 | 100 | 108250 | 100 | | 22945 | 100 | 36998 | 100 | 59943 | 100 |
|  |  |  |  |  |  |  | |  |  |  |  |  |  |

Abbreviations: BTOS, Broad Type of Service; ENZA, enzalutamide; NSAA, nonsteroidal anti-androgen; MBS = Medicare Benefits Schedule

Footnotes:

^a^ including oral & maxillofacial services and preventive services

^b^ Based on 504 consenting Australian participants with linked MBS claims

**Table S3.2 MBS costs^a^ by Broad Type of Service (BTOS) classification and study status**

| Broad Type of Service (BTOS) | On study treatment | | | | | | Off study treatment | | | | | |
| --- | --- | --- | --- | --- | --- | --- | --- | --- | --- | --- | --- | --- |
|  | ENZA | | NSAA | | All | | ENZA | | NSAA | | All | |
|  | Cost (AUD$, 2023) | % | Cost (AUD$, 2023) | % | Cost (AUD$, 2023) | % | Cost (AUD$, 2023) | % | Cost (AUD$, 2023) | % | Cost (AUD$, 2023) | % |
|  |  |  |  |  |  |  |  |  |  |  |  |  |
| Diagnostic Imaging Services | $759,000 | 20 | $677,000 | 27 | $1,436,000 | 23 | $423,000 | 24 | $736,000 | 26 | $1,159,000 | 25 |
| Diagnostic Procedures and Investigations | $89,000 | 2 | $57,000 | 2 | $146,000 | 2 | $18,000 | 1 | $24,000 | 1 | $42,000 | 1 |
| Pathology Services | $576,000 | 15 | $374,000 | 15 | $950,000 | 15 | $190,000 | 11 | $322,000 | 11 | $512,000 | 11 |
| Professional Attendances | $1,483,000 | 39 | $863,000 | 34 | $2,346,000 | 37 | $499,000 | 28 | $770,000 | 27 | $1,269,000 | 28 |
| Therapeutic Procedures | $749,000 | 20 | $516,000 | 20 | $1,265,000 | 20 | $650,000 | 36 | $927,000 | 33 | $1,577,000 | 34 |
| Miscellaneous Services ^b^ | $100,000 | 3 | $47,000 | 2 | $147,000 | 2 | $19,000 | 1 | $36,000 | 1 | $55,000 | 1 |
|  |  |  |  |  |  |  |  |  |  |  |  |  |
| Total | $3,756,000 | 100 | $2,534,000 | 100 | $6,290,000 | 0 | $1,799,000 | 100 | $2,815,000 | 100 | $4,614,000 | 0 |
|  |  |  |  |  |  |  |  |  |  |  |  |  |

Abbreviations: AUD$, Australian dollar; BTOS, Broad Type of Service; ENZA, enzalutamide; NSAA, nonsteroidal anti-androgen; MBS = Medicare Benefits Schedule

Footnotes:

^a^ Based on 504 consenting Australian participants with linked MBS claims

^b^ included oral & maxillofacial services and preventive services

**Table S4.1 Prescribed medicines claims^a^ by drug classification and study status**

| Anatomic Therapeutic Chemical classification (1st level) | On study treatment | | | | | | Off study treatment | | | | | |
| --- | --- | --- | --- | --- | --- | --- | --- | --- | --- | --- | --- | --- |
|  | ENZA | | NSAA | | All | | ENZA | | NSAA | | All | |
|  | Claims | % | Claims | % | Claims | % | Claims | % | Claims | % | Claims | % |
| A: Alimentary tract and metabolism | 7811 | 15.9 | 3966 | 11.9 | 11777 | 14.3 | 2229 | 16.8 | 3524 | 14.4 | 5753 | 15.3 |
| B: Blood and blood forming organs | 1865 | 3.8 | 1535 | 4.6 | 3400 | 4.1 | 585 | 4.4 | 861 | 3.5 | 1446 | 3.8 |
| C: Cardiovascular system | 18906 | 38.5 | 9582 | 28.8 | 28488 | 34.6 | 2913 | 21.9 | 5589 | 22.9 | 8502 | 22.5 |
| D: Dermatologicals | 292 | 0.6 | 174 | 0.5 | 466 | 0.6 | 121 | 0.9 | 95 | 0.4 | 216 | 0.6 |
| G: Genito urinary system & sex hormones | 394 | 0.8 | 67 | 0.2 | 461 | 0.6 | 115 | 0.9 | 118 | 0.5 | 233 | 0.6 |
| H: Systemic hormonal preparations | 482 | 1.0 | 357 | 1.1 | 839 | 1.0 | 936 | 7.0 | 1371 | 5.6 | 2307 | 6.1 |
| J: Antiinfective for systemic use | 1946 | 4.0 | 1095 | 3.3 | 3041 | 3.7 | 509 | 3.8 | 729 | 3.0 | 1238 | 3.3 |
| L: Antineoplastic & immunomodulating agents | 5389 | 11.0 | 11021 | 33.2 | 16410 | 19.9 | 1943 | 14.6 | 4586 | 18.8 | 6529 | 17.3 |
| M: Musculo-skeletal system | 2442 | 5.0 | 872 | 2.6 | 3314 | 4.0 | 785 | 5.9 | 1608 | 6.6 | 2393 | 6.3 |
| N: Nervous system | 7022 | 14.3 | 3047 | 9.2 | 10069 | 12.2 | 2882 | 21.7 | 4978 | 20.4 | 7860 | 20.8 |
| P: Antiparasitic products, insecticides & repellents | 1 | 0.0 | 4 | 0.0 | 5 | 0.0 | 5 | 0.0 | 0 | 0.0 | 5 | 0.0 |
| R: Respiratory system | 1248 | 2.5 | 793 | 2.4 | 2041 | 2.5 | 137 | 1.0 | 486 | 2.0 | 623 | 1.7 |
| S: Sensory organs | 1211 | 2.5 | 718 | 2.2 | 1929 | 2.3 | 143 | 1.1 | 328 | 1.3 | 471 | 1.2 |
| V: Various | 43 | 0.1 | 6 | 0.0 | 49 | 0.1 | 5 | 0.0 | 129 | 0.5 | 134 | 0.4 |
|  |  |  |  |  |  |  |  |  |  |  |  |  |
| Total | 49052 | 100 | 33237 | 100 | 82289 | 100 | 13308 | 100 | 24402 | 100 | 37710 | 100 |
|  |  |  |  |  |  |  |  |  |  |  |  |  |

Abbreviations: ENZA, enzalutamide; NSAA, nonsteroidal anti-androgen

Footnotes:

^a^ Based on 500 consenting Australian participants with linked PBS claims

**Table S4.2 Prescribed medicines costs ^a^ by drug classification and study status**

| Anatomic Therapeutic Chemical classification (1st level) | On study treatment | | | | | | Off study treatment | | | | | |
| --- | --- | --- | --- | --- | --- | --- | --- | --- | --- | --- | --- | --- |
|  | ENZA | | NSAA | | All | | ENZA | | NSAA | | All | |
|  | Cost (AUD$, 2023) ^b^ | % | Cost (AUD$, 2023) | % | Cost (AUD$, 2023) | % | Cost (AUD$, 2023) | % | Cost (AUD$, 2023) | % | Cost (AUD$, 2023) | % |
| A: Alimentary tract & metabolism | $225,000 | 3 | $98,000 | 2 | $323,000 | 3 | $62,000 | 2 | $76,000 | 1 | $138,000 | 1 |
| B: Blood and blood forming organs | $108,000 | 2 | $86,000 | 2 | $194,000 | 2 | $48,000 | 1 | $60,000 | 1 | $108,000 | 1 |
| C: Cardiovascular system | $227,000 | 3 | $111,000 | 2 | $338,000 | 3 | $39,000 | 1 | $87,000 | 1 | $126,000 | 1 |
| D: Dermatologicals | $5,000 | 0 | $4,000 | 0 | $9,000 | 0 | $4,000 | 0 | $2,000 | 0 | $6,000 | 0 |
| G: Genito urinary system & sex hormones | $9,000 | 0 | $1,000 | 0 | $10,000 | 0 | $3,000 | 0 | $3,000 | 0 | $6,000 | 0 |
| H: Systemic hormonal preparations | $13,000 | 0 | $2,000 | 0 | $15,000 | 0 | $8,000 | 0 | $10,000 | 0 | $18,000 | 0 |
| J: Antiinfective for systemic use | $27,000 | 0 | $202,000 | 4 | $229,000 | 2 | $10,000 | 0 | $9,000 | 0 | 19,000 | 0 |
| L: Antineoplastic & immunomodulating agents | $5,442,000 | 79 | $4,076,000 | 84 | $9,518,000 | 81 | $3,343,000 | 87 | $10,004,000 | 91 | $13,347,000 | 90 |
| M: Musculo-skeletal system | $337,000 | 5 | $133,000 | 3 | $470,000 | 4 | $234,000 | 6 | $593,000 | 5 | $827,000 | 6 |
| N: Nervous system | $177,000 | 3 | $75,000 | 2 | $252,000 | 2 | $97,000 | 3 | $137,000 | 1 | $234,000 | 2 |
| P: Antiparasitic products, insecticides & repellents | - | 0 | - | 0 | - | 0 | $1,000 | 0 | - | . | $1,000 | 0 |
| R: Respiratory system | $111,000 | 2 | $37,000 | 1 | $148,000 | 1 | $6,000 | 0 | $23,000 | 0 | $29,000 | 0 |
| S: Sensory organs | $173,000 | 3 | $24,000 | 0 | $197,000 | 2 | $2,000 | 0 | $19,000 | 0 | $21,000 | 0 |
| V: Various | $4,000 | 0 | - | 0 | $4,000 | 0 | - | 0 | $10,000 | 0 | $10,000 | 0 |
|  |  |  |  |  |  |  |  |  |  |  |  |  |
| All | $6,858,000 | 100 | $4,849,000 | 100 | $11,707,000 | 100 | $3,857,000 | 100 | $11,033,000 | 100 | $14,890,000 | 100 |
|  |  |  |  |  |  |  |  |  |  |  |  |  |

Abbreviations: AUD, Australian dollar; ENZA, enzalutamide; NSAA, nonsteroidal anti-androgen

Footnotes:

^a^ Based on 500 consenting Australian participants with linked PBS claims

^b^ rounded to the nearest AUD$1,000

Table S5.1. Mean values, standard deviations, and estimated distribution parameters used in the probabilistic sensitivity analysis (PSA)

| Parameter |  | Enzalutamide | NSAA | Distribution | Distribution parameter | Enzalutamide | NSAA |
| --- | --- | --- | --- | --- | --- | --- | --- |
| Study drug | Mean | 150254 | 1648 | Gamma | α (Shape) | 4.109 | 2.141 |
|  | SD | 74121.69 | 1126.19 |  | θ (Scale) | 36564.916 | 769.602 |
| ICU stays | Mean | 1,008 | 253 | Gamma | α (Shape) | 0.3775^1^ | 0.1712^1^ |
|  | SD | 1,640.73 | 611.41 |  | θ (Scale) | 2,670.63 | 1,477.56 |
| HDU stays | Mean | 343 | 116 | Gamma | α (Shape) | 0.1343^1^ | 0.0928^1^ |
|  | SD | 935.99 | 380.73 |  | θ (Scale) | 2,554.17 | 1,249.62 |
| Ward stays | Mean | 9,278 | 5,264 | Gamma | α (Shape) | 3.132 | 2.667 |
|  | SD | 5,242.56 | 3,223 |  | θ (Scale) | 2,962.17 | 1973.50 |
| ED presentations | Mean | 455 | 319 | Gamma | α (Shape) | 7.520 | 4.664 |
|  | SD | 165.93 | 147.71 |  | θ (Scale) | 60.51 | 68.40 |
| MBS on treatment | Mean | 11196 | 8891 | Gamma | α (Shape) | 117.320 | 60.323 |
|  | SD | 1,033.66 | 1,144.75 |  | θ (Scale) | 95.432 | 147.391 |
| MBS off treatment | Mean | 5151 | 9461 | Gamma | α (Shape) | 4.689 | 17.878 |
|  | SD | 2,378.65 | 2,237.56 |  | θ (Scale) | 1098.423 | 529.191 |
| PBS on treatment | Mean | 20830 | 17619 | Gamma | α (Shape) | 573.917 | 46.312 |
|  | SD | 869.49 | 2,589.02 |  | θ (Scale) | 36.294 | 380.443 |
| PBS off treatment | Mean | 11710 | 39393 | Gamma | α (Shape) | 5.947 | 53.660 |
|  | SD | 4,801.86 | 5,377.68 |  | θ (Scale) | 1969.074 | 734.126 |
| EQ-5D-5L utility -on treatment | Mean | 0.78 | 0.81 | Beta ^2^ | α (Shape) | 1.887 | 2.547 |
|  | SD | 0.2239 | 0.1927 |  | β (Shape) | 0.531 | 0.597 |

^1^ For Gamma variables with shape parameter α < 0.5 (ICU stay, HDU stay), a Devroye transformation (2) for Gamma random variate generation was applied to improve numerical stability during simulation.

^2^ Parameter validity for the Beta distributions in both treatment arms was verified.

Table S5.2. PSA summary statistics: Enzalutamide vs. NSAA based on 100,000 Monte Carlo simulations

|  | Average | Standard deviation | Stand error | Lower 95%CI | Upper 95% |
| --- | --- | --- | --- | --- | --- |
| **Cost** |  |  |  |  |  |
| 5-year cost per participant randomized to enzalutamide (AUD$) | $209,855 | $74,273 | $235 | $209,395 | $210,316 |
| 5-year cost per participant randomized to NSAA (AUD$) | $82,962 | $7,378 | $23 | $82,916 | $83,008 |
| Incremental cost (AUD$) | $126,893 | $74,649 | $236 | $126,431 | $127,356 |
| **QALY** |  |  |  |  |  |
| 5-year QALY per participant randomized to enzalutamide | 3.02 | 0.78 | 0.00 | 3.01 | 3.02 |
| 5-year QALY per participant randomized to NSAA | 2.87 | 0.48 | 0.00 | 2.86 | 2.87 |
| Incremental QALY | 0.15 | 0.92 | 0.00 | 0.14 | 0.16 |

**References**

1.	Briggs AH, Weinstein MC, Fenwick EA, Karnon J, Sculpher MJ, Paltiel AD, et al. Model parameter estimation and uncertainty: a report of the ISPOR-SMDM Modeling Good Research Practices Task Force--6. Value Health. 2012;15(6):835-42.

2.	Devroye L. Non-Uniform Random Variate Generation. New York, NY: Springer-Verlag; 1986.

Figure S1. Location of ENZAMET trial sites by country

Footnote:

The map was created using MapChart: https://www.mapchart.net/world.html
